# A survey of medicinal plants used by the Deb barma clan of the Tripura tribe of Moulvibazar district, Bangladesh

**DOI:** 10.1186/1746-4269-10-19

**Published:** 2014-02-06

**Authors:** Mohammad Humayun Kabir, Nur Hasan, Md Mahfuzur Rahman, Md Ashikur Rahman, Jakia Alam Khan, Nazia Tasnim Hoque, Md Ruhul Quddus Bhuiyan, Sadia Moin Mou, Rownak Jahan, Mohammed Rahmatullah

**Affiliations:** 1 Department of Pharmacy, University of Development Alternative, Dhanmondi, Dhaka 1209, Bangladesh; 2 Faculty of Life Sciences, University of Development Alternative, House No. 78, Road No. 11A (new), Dhanmondi, Dhaka 1209, Bangladesh

**Keywords:** Indigenous knowledge, Allopathic medicine, Ethnomedicine, Bangladesh

## Abstract

**Background:**

The number of tribes present within Bangladesh has been estimated to approximate one hundred and fifty. Information on traditional medicinal practices, particularly of the smaller tribes and their clans is lacking. It was the objective of the study to document the tribal medicinal practices of the Deb barma clan of the Tripura tribe, which clan can be found residing in Dolusora Tripura Palli of Moulvibazar district of Bangladesh. A further objective was to determine the extent of the community households who still prefer traditional treatment to other forms of treatment, particularly allopathic treatment.

**Methods:**

Interviews of the tribal healer and the tribal community regarding their ethnomedicinal practices were carried out with the help of a semi-structured questionnaire and the guided field-walk method. All together 67 clan members were interviewed including the Headman, tribal healer, 19 Heads of households and 46 other adult members of the clan. Information on number of members of household, their age, gender, educational status, occupation of working household members and preferred mode of treatment was obtained through the semi-structured questionnaire. In the guided field-walk method, the healer took the interviewers on field-walks through areas from where he collected his medicinal plants, pointed out the plants, and described their uses.

**Results:**

The clan had a total of 135 people distributed into 20 households and had only one traditional healer. Use of medicinal plants, wearing of amulets, and worship of the evil god ‘Bura debta’ constituted the traditional medicinal practices of the clan for treatment of diseases. The healer used a total of 44 medicinal plants distributed into 34 families for treatment of various ailments like pain, coughs, cold, gastrointestinal disorders, cuts and wounds, diabetes, malaria, heart disorders, and paralysis.

**Conclusions:**

Available scientific reports validate the use of a number of plants by the traditional healer. A number of the plants used by the clan healer had reported similar uses in Ayurveda, but differ considerably in their therapeutic uses from that reported for other tribes in Bangladesh. The present survey also indicated that in recent years the Deb barma clan members are inclining more towards allopathic medicine.

## Background

Since the advent of human beings, it is very much possible that they were afflicted with diseases and in course of time started using various ingredients including plants, animals, insects, or minerals for treatment. It has been reported that human beings were aware of the medicinal properties of plants even around 5,000 years ago [1]. Since then, even after the introduction of modern or allopathic medicine, medicinal plants have played a vital role in the traditional medicinal systems of many countries, as well as being the sources of many modern drugs. Indeed, it has been reported that a number of important allopathic drugs like aspirin, atropine, ephedrine, digoxin, morphine, quinine, reserpine, artemisinin and tubocurarine have been discovered through close observations of traditional medicinal practices of indigenous peoples [2].

Bangladesh is home to a number of tribes or indigenous communities. Latest ethnographic research suggests that the number of tribes within the country approximates 150 instead of the previously estimated about a dozen tribes [3]. Most of the indigenous communities and particularly the smaller ones (i.e. communities whose population is below 500 persons) are on the verge of disappearance because of decline in population, loss in tribal habitat, or because of merging with the mainstream Bengali-speaking population. As a result, the culture and knowledge possessed by these tribes are also fast disappearing, including their traditional medicinal practices. Adequate documentation of such knowledge, and especially traditional medicinal practices, is important because tribal medicinal practitioners or healers through long association with plants around their vicinity have acquired quite extensive knowledge on the medicinal properties of these various plant species. Notably, tribal medicinal knowledge is usually passed from one generation to the next through members of the family or persons serving as apprentices to the practitioner. Thus such tribal medicinal knowledge reflects knowledge acquired and accumulated over centuries and even possibly millennia.

Scientists as well as general human beings can gain a considerable amount of information from adequate documentation of tribal medicinal practices. Adequate documentation can not only indicate the possible therapeutic values of any given plant species, but also provide scientists with a general background on the basis of which they can study the plant species for isolation of bioactive constituents. Documentation of medicinal plants used in the country in various traditional medicinal systems existing within the country can also spur conservation efforts of these plants, many of which are getting endangered through continuous deforestation and increase of human habitat. Bangladesh has several ancient medicinal systems, which are still in practice. Although to a certain extent, some of these various traditional medicinal systems influence and overlap one another, these systems can broadly be classified as Ayurveda, Unani, homeopathy, and folk and tribal medicine. Of these systems, Ayurveda, Unani, and folk and tribal medicinal systems rely quite extensively on medicinal plants, which are used in simple or complex formulations for treatment of different diseases. Among these systems, Ayurveda and Unani are more organized and each system has their own well-established formulary, and practitioners who graduate from Ayurveda or Unani colleges in the country. On the contrary, folk medicinal practitioners (known as Kavirajes or Vaidyas) and tribal medicinal practitioners each have their own field of expertise and unique repertoire of medicinal plants, which can vary greatly from tribe to tribe and between individual Kavirajes of even the same area.

Towards building up a comprehensive database of medicinal plants of the country and their traditional uses, we had been interviewing and documenting the traditional medicinal practices of folk and tribal medicinal practitioners for a number of years [4-11]. The Tripura (also known as Tripuri, Tiprah or Tipperah) tribe is one such indigenous community in Bangladesh, whose various clans can be found in the Chittagong and Sylhet Divisions in the southeast and northeast parts, respectively, of the country. The various clans of the Tripura tribe include Deb barma (also known as Tiprah), Reang or Bru, Jamatia, Koloi, Noatia, Murasing, Halam, Harbang, and Uchoi. We have previously documented the ethnomedicinal practices of the Harbang clan of the Tripura tribal community residing in Chittagong Division of Bangladesh [12].

**Figure 1:**
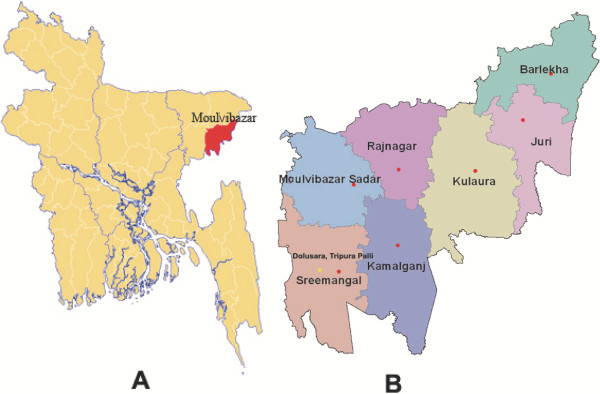
**Map of Bangladesh showing survey site area. (A)** Bangladesh with Moulvibazar district highlighted in red and **(B)** Dolusora Tripura Palli (site of survey, indicated with a yellow dot) in Moulvibazar district.

The objective of the present study was to document the ethnomedicinal practices of the Deb barma clan of the Tripura tribe residing in Dolusora Tripura Palli, which falls within Moulvibazar district in Sylhet Division of Bangladesh (Figure 1). The whole clan consisted of 20 households and had a total population of 135. They resided in a single village named Dolusora Tripura Palli, the Palli name indicating village or area of residence. The Palli itself fell within Moulvibazar district of Sylhet Division in the northeastern part of Bangladesh. The Headman, namely, Mahendra Lal Deb barma of Tripura Palli is considered a renowned person among the Deb barma clan members. The clan had only one tribal healer, named Shorbanando Tripura (otherwise also known as Shorbanando Deb barma). Every individual household had a person acting as the Head of the household. The Head of household was in all cases the most elderly but still active member of the house irrespective of gender. A secondary objective was to conduct a survey among this tribal community to determine the extent of preference for tribal medicine versus allopathic medicine within members of the community.

The Deb barma clan claimed themselves to be Hindus. They worshipped the Hindu god ‘Shiva’ and the Hindu goddess ‘Kali’. However, they mentioned to the interviewers that they also worshipped fourteen other gods and goddesses of their own. The Headman mentioned that once upon a time all Tripura clans were animists, but now all clans have become Hindus but still retained many of their animist traditions. The Deb barma clan also mentioned that they believe in evil spirits and demons. Among the gods and goddesses that the Deb barma clan believed in was the god whom the Headman referred to as ‘Bura debta’, signifying old god. Bura Debta was considered an evil god by the Deb Barma clan and it was considered that the clan must always appease him through ‘pujas’ (worship) and ‘archanas’ (offerings). As a result, the Deb barma clan performs two types of pujas per year in which offerings are made to satisfy Bura debta.

It was mentioned by the Deb barmas that disease occurs to a person if Bura debta gets angry for some reason and curses that person. However, the Deb barmas also said that they believe that diseases can be caused by evil spirits and demons who reside in the forest areas. When evil spirits cause disease(s), according to them, it is known as ‘upuri’ (paranormal diseases). Such paranormal diseases (like being possessed by ‘genies’ or ‘ghosts’) are due to black magic, and the clan believes that diseases caused by black magic can be cured through the interventions of a Tantrik, i.e. a person who is knowledgeable in and can perform black magic. On the other hand, a traditional medicinal healer (Kaviraj) can cure them from diseases caused through the wrath of Bura debta. The Kaviraj can also be a Tantrik. Thus Deb barma clan traditional healing is a mixture of medicinal plant formulations received from the Kaviraj, wearing of amulets as prescribed by the Kaviraj, pujas of Bura debta, as well as counter-black magic performed by a Tantrik. However, at present, the Deb barma clan did not have any specialized Tantriks among them; they only had one traditional medicinal healer, who also took care of problems like being possessed by ‘genies’ or ‘ghosts’.

Since ethnomedicinal surveys of various tribes and folk medicinal practitioners are still at an early stage in Bangladesh, the primary objective of the present study was to document the hitherto unreported traditional medicinal practices of the Deb barma clan of the Tripura tribe. Secondary objectives were (I) to determine whether such medicinal practices have been influenced by the most ancient form of traditional medicine in Bangladesh, namely Ayurveda, (II) to determine whether the use of medicinal plants by the Deb barma healer could be scientifically validated on the basis of available scientific studies on pharmacological properties of any specific plant, (III) to analyze comparative uses of the medicinal plants by the Deb barma healer with other reported ethnomedicinal uses from Bangladesh, and (IV) to determine to what extent individual households of the Deb barma clan are still utilizing the services of their traditional healer or in the present age switching to other modes of treatment like allopathic medicine.

## Methods

The survey was conducted between August 2012 and May 2013 at Dolusora Tripura Palli (Palli meaning village). A number of visits (8, each visit lasting 2-4 days) were made to the Deb barma clan to build up rapport with the Headman, healer, and members of the Deb barma clan. Prior Informed Consent was obtained from the Headman, healer, Heads of households and adult members of the clan to interview them as to their traditional medicinal practices (healer) and to their choice of traditional medicine versus allopathic medicine (rest of the persons interviewed). Essentially, the Headman, healer and Heads of households provided the answers with other adult members of households concurring with the opinions of the Head of each respective household. As such, although 67 members were interviewed, the actual number of actively responding members were 21, comprising of 1 healer [male], 1 Headman [also the Head of a household (male)], and 19 other Heads of households [13 males and 6 females]. With the exception of 2 Heads of households (both males) who mentioned their ages as 43 and 47 years, the rest of the Heads of households (including the Headman) and the healer were above 50 years old. Allopathic medicine was provided to them by an allopathic doctor, who belonged to a NGO (non-Governmental organization), which organization worked in the general area of Srimangal (where the Tripura Palli was located) among the rural people, including both mainstream Bengali-speaking people, as well as the Deb barma clan of the Tripuras. Actual interviews of all persons, and especially the traditional healer were conducted with the help of a semi-structured questionnaire and the guided field-walk method of Martin [13] and Maundu [14]. Through the semi-structured questionnaire, information was obtained from the healer and other clan members as to their age, gender, educational status, occupation and monthly income, number of family members, food habits, what they thought of diseases as well as medical preferences. The healer was further queried with the help of the semi-structured questionnaire as to plants used, disease(s) treated, mode of collection and preservation of plants, formulations, mode of administration, and any precautions which needed to be followed during medication period together with any other details which the healer wanted to provide. Briefly, in the guided field-walk method, the healer took the interviewers on guided field-walks through areas from where he collected his medicinal plants, pointed out the plants, and mentioned their use(s).

The adult clan members as well as a few young members (under 18 years of age) worked as agricultural laborers in a nearby tea estate, where the female members were engaged in plucking tea leaves, and the male members engaged in maintaining tea gardens (including plantation, fertilization, weeding, and watering). The socio-economic status of the clan households were poor and every household reported that their daily income was below the poverty level, which has been defined by the Government of Bangladesh as less than US$ 1 per day. The adult clan members were illiterate; a few children attended schools but were quickly taken out of school before they reached Grade VI so as to work in the tea estate and augment the family income. Housing and conditions of living were in a primitive state with poor hygienic conditions and lack of proper sanitation facilities.

It was observed that all plants used by the healer were collected within Dolusora Tripura Palli or from adjoining sites, i.e. within 10 km of Dolusora Tripura Palli. Plants or plant parts were collected free of cost. Most plants were perennial, i.e. available throughout the year. If any plant part was not available (e.g. fruits) throughout the year, the healer used dried fruits as in the cases of *Phyllanthus emblica*, *Terminalia bellirica*, and *Terminalia chebula. Allium sativum*, another plant used by the healer is also an annual plant, but bulbs of this plant (garlic) were used, which were available throughout the year in the dried form. However, if any plant or plant part necessary for a formulation was not found, the healer did not treat the disease that the plant or plant part was intended to be used. Plant specimens were photographed and collected on the spot. They were then pressed, dried and brought back to Dhaka. Identification of plants was done by Mr. Manjur-Ul-Kadir Mia, ex-Curator and Principal Scientific Officer of the Bangladesh National Herbarium. Voucher specimens were deposited with the Medicinal Plant Collection Wing of the University of Development Alternative. Interviews were conducted in the Bengali (Bangla) language; all Tripura community members were found to be quite fluent with this language of the mainstream population through long-term association with the mainstream people. The Bangladesh Government has opened a Bangla-medium primary school near the Tripura Palli and several students attended or are still attending the school.

## Results

### Medicinal plants and diseases treated by the tribal healer

Of the twenty households of the Deb barma clan, the Head of household of 14 families were males and that of 6 families were females, these persons being the most elderly but still active (i.e. working) members of the house. The traditional medicinal healer mentioned that he uses a total of 44 medicinal plants for treatment of a variety of ailments. These plants were distributed into 34 families and are shown in Table 1. The various ailments treated by the healer included malaria, skin infections, tuberculosis, respiratory disorders, bleeding from external cuts and wounds, chest pain, gastrointestinal disorders, rheumatic pain, burning sensations during urination, bone fracture, snake bite, toothache, headache, bleeding from gums, paralysis, skin disorders, helminthiasis, chicken pox, diabetes, jaundice, eye disorders, weakness, and being possessed by ‘genies’ or ‘ghosts’. Two plants parts (bulb of *Crinum latifolium* along with bulb of *Allium sativum*) were also used in combination for treatment of bloating in cattle (see Serial Number 5), and one plant, *Scoparia dulcis*, used to prepare wine (See Serial Number 37). In this context, it is interesting to note that the clan healer advised wearing an amulet containing the plant, *Asparagus racemosus*, for all diseased persons, irrespective of the disease or other medicinal plants used. This particular plant was considered to have special magical properties that appease Bura debta.

**Table 1:** Medicinal plants and formulations of the Deb barma clan healer

**Serial number**	**Scientific name (voucher specimen number)**	**Family name (perennial/annual)**	**Local name (english name)**	**Parts used**	**Disease, symptoms, formulations, and administration**
1	*Andrographis paniculata* Nees MPCW-UODA 3032/2013	Acanthaceae (Annual)	Boner kalomegh (Green chirayta)	Leaf	Malaria. Juice obtained from crushed leaves is orally taken.
2	*Justicia adhatoda* L. MPCW-UODA 3033/2013	Acanthaceae (Perennial)	Shada bashok (Malabar nut)	Leaf	Skin infections. Young leaves are made into a paste and applied as poultice over infected area.
					Tuberculosis. Juice obtained from crushed leaves is orally taken.
3	*Justicia gendarussa* Burm. f. MPCW-UODA 3036/2013	Acanthaceae (Perennial)	Kala bashok (Black adusa)	Leaf, stem	Coughs. Leaves are boiled in water. ½ poa (local measure, 4 poas approximate 1 kg) of the decoction is taken orally thrice daily.
					Malaria. Crushed leaves and stems are boiled in water followed by drinking the water daily till cure.
4	*Aerva sanguinolenta* (L.) Blume MPCW-UODA 3038/2013	Amaranthaceae (Perennial)	Iodine pata (Kapok bush)	Leaf	To stop bleeding from external cuts and wounds. Leaves are crushed between the fingers and applied to cuts and wounds.
5	*Crinum latifolium* L. MPCW-UODA 3039/2013	Amaryllidaceae (Perennial)	Gruna, bon roshun (Milk and wine lily)	Bulb	Chest pain (symptoms: pain in the chest area, usually external muscle pain). Juice obtained from crushed bulb is taken orally.
					Bloating in cattle. Bulbs of *Crinum latifolium* are fed to cattle along with bulbs of *Allium sativum*.
6	*Centella asiatica* (L.) Urb. MPCW-UODA 3040/2013	Apiaceae (Perennial)	Thankuni (Asiatic pennywort)	Leaf, stem	Stomach disorders (stomach pain, flatulency). Juice obtained from crushed leaves and stems is taken orally.
7	*Alstonia scholaris* (L.) R. Br. MPCW-UODA 3044/2013	Apocynaceae (Perennial)	Chaitan (Blackboard tree)	Leaf sap	Whitish layer formation on tongue. Leaf sap is mixed with honey and applied to tongue.
8	*Colocasia esculenta* (L.) Schott. MPCW-UODA 3046/2013	Araceae (Perennial)	Fan kochu (Taro)	Tuber	Rheumatic pain. Dried and powdered tubers are taken orally.
9	*Borassus flabellifer* L. MPCW-UODA 3047/2013	Arecaceae (Perennial)	Taal (Palmyra palm)	Sap	See *Hyptis suaveolens*.
10	*Asparagus racemosus* Willd. MPCW-UODA 3048/2013	Asparagaceae (Perennial)	Shotomul (Indian asparagus)	Whole plant	All diseases. Whole plant is put in an amulet which is to be tied by the patient around the waist with a piece of thread and worn till the disease is cured.
11	*Ageratum conyzoides* L. MPCW-UODA 3049/2013	Asteraceae (Perennial)	Ujaru (Mexican ageratum)	Root	Stomach disorders. Juice obtained from crushed roots is orally taken.
12	*Garcinia cowa* Roxb. MPCW-UODA 3050/2013	Clusiaceae (Perennial)	Kau fol (Cowa-mangosteen)	Fruit	Coughs, cold. Fruits are eaten.
13	*Terminalia arjuna* (Roxb. ex DC.) Wight & Arn. MPCW-UODA 3053/2013	Combretaceae (Perennial)	Arjun (Arjun tree)	Bark	Chest pain due to heart disorders (symptoms: chest pain more so when walking or working, feeling of constriction of heart, breathlessness), burning sensations during urination, bone fracture.
					Bark is boiled in water followed by drinking the water for chest pain and burning sensations during urination. Paste prepared from bark of *Terminalia arjuna* and clove of *Allium sativum* is applied as poultice over the fractured area.
14	*Terminalia bellirica* (Gaertn.) Roxb. MPCW-UODA 3055/2013	Combretaceae (Perennial)	Bohera (Beleric myrobalan)	Fruit, leaf	See *Phyllanthus emblica*.
15	*Terminalia chebula* Retz. MPCW-UODA 3056/2013	Combretaceae (Perennial)	Horitoki (Chebulic myrobalan)	Fruit, leaf	See *Phyllanthus emblica*.
16	*Sansevieria hyacinthoides* (L.) Druce MPCW-UODA 3058/2013	Dracaenaceae (Perennial)	Dudh raj (Iguana tail)	Leaf	Snake bite, snake repellent. Juice obtained from crushed leaf is applied to snake-bitten area. Leaves are kept in rooms to repel snakes.
17	*Jatropha curcas* L. MPCW-UODA 3060/2013	Euphorbiaceae (Perennial)	Kangala (Physic nut)	Leaf	Tooth ache. Leaves are chewed and the leaf juice swallowed.
18	*Phyllanthus emblica* L. MPCW-UODA 3061/2013	Euphorbiaceae (Perennial)	Amloki (Indian gooseberry)	Fruit, leaf	Bleeding from gums, loss of appetite, headache. Unripe fruits are chewed to stop bleeding from gums. Dried fruits of *Phylanthus emblica*, *Terminalia bellirica* and *Terminalia chebula* are soaked in water followed by drinking the water to increase appetite. Fruits of *Phyllanthus emblica* and wood of *Santalum album* are made into a paste with rose water and applied to the forehead for headache.
					Paralysis. Juice obtained from a combination of crushed leaves of *Phyllanthus emblica*, *Terminalia bellirica* and *Terminalia chebula* was massaged on the paralyzed area.
19	*Phyllanthus reticulatus* Poir. MPCW-UODA 3062/2013	Euphorbiaceae (Perennial)	Khaukora (Sour grapes)	Stem	Diarrhea in children. Stems are twisted and tied loosely around the neck of children.
20	*Senna alata* (L.) Roxb. MPCW-UODA 3063/2013	Fabaceae (Perennial)	Daud pata (Candle bush)	Leaf	Eczema. Juice obtained from crushed leaves is topically applied.
21	*Hyptis suaveolens* (L.) Poit. MPCW-UODA 3064/2013	Lamiaceae (Perennial)	Tukma (Pignut)	Fruit	Physical weakness, sense of hotness in head. Powdered fruits of *Hyptis suaveolens* are mixed with tal mishri (sugar obtained from sap of *Borassus flabellifer*) and taken orally.
22	*Leucas aspera* (Willd.) Link MPCW-UODA 3065/2013	Lamiaceae (Annual)	Deo kolosh (Common leucas)	Flower	Coughs in infant. Juice obtained from crushed flower is mixed with mother’s milk and orally administered.
23	*Ocimum gratissimum* L. MPCW-UODA 3066/2013	Lamiaceae (Perennial)	Shada tulsi (African basil)	Leaf	Rheumatic pain, coughs, mucus. A piece of cloth is soaked with juice obtained from crushed leaves is tied to areas affected by rheumatic pain. Juice obtained from crushed leaves is taken with honey for coughs and mucus.
24	*Allium sativum* L. MPCW-UODA 3067/2013	Liliaceae (Annual)	Roshun	Bulb	See *Crinum latifolium*.
					See *Terminalia arjuna*.
25	*Lawsonia inermis* L. MPCW-UODA 3070/2013	Lythraceae (Perennial)	Mehedi (Henna)	Leaf	Cuts and wounds, cracked skin, diabetes, burning sensations during urination. Paste of leaves is applied to cuts and wounds or cracked skin. Juice obtained from crushed leaves is orally taken for diabetes and burning sensations during urination.
26	*Melastoma malabathricum* L. MPCW-UODA 3072/2013	Melastomataceae (Perennial)	Luiki (Malabar melastome)	Leaf	To stop bleeding from external cuts and wounds. Crushed leaves are applied as poultice over cuts and wounds.
27	*Azadirachta indica* A. Juss. MPCW-UODA 3073/2013	Meliaceae (Perennial)	Neem (Nim tree)	Leaf, bark	Itches, ringworm. Leaves of *Azadirachta indica* are boiled in water and then made into a paste with rhizomes of *Curcuma longa*. The paste is applied to affected areas of skin after taking a bath.
					Helminthiasis. Powdered bark is taken orally with table salt.
					Chicken pox. Paste of leaves of *Azadirachta indica* and rhizomes of *Curcuma longa* are applied to pustules.
28	*Ficus hispida* L. MPCW-UODA 3074/2013	Moraceae (Perennial)	Shada dumur (Hairy fig)	Fruit	Diabetes. Dried and powdered fruits are eaten regularly.
29	*Moringa oleifera* Lam. MPCW-UODA 3075/2013	Moringaceae (Perennial)	Sajna (Horseradish tree)	Leaf, stem	Jaundice. Juice obtained from crushed leaves and stems is taken orally.
30	*Psidium guajava* L. MPCW-UODA 3077/2013	Myrtaceae (Perennial)	Peyara (Guava)	Leaf bud, fruit	Tooth infections, loss of appetite. Leaf buds are boiled in water followed by gargling with the water for tooth infections. Fruits are chewed and eaten to stimulate appetite.
31	*Plumbago indica* L. MPCW-UODA 3079/2013	Plumbaginaceae (Perennial)	Agun pata (Scarlet leadwort)	Leaf	Pain. Leaves are fried in any oil and ghee (clarified butter) and massaged on the painful areas.
32	*Persicaria glabra* (Willd.) M. Gómez MPCW-UODA 3080/2013	Polygonaceae (Perennial)	Kathali kot (Denseflower knotweed)	Leaf sap	Conjunctivitis. Leaf sap is applied to eyes.
33	*Eichhornia crassipes* (Mart.) Solms MPCW-UODA 3084/2013	Pontederiaceae (Perennial)	Pana kochuri (Common water hyacinth)	Root	Feeling of hotness in head. Roots are soaked in water and kept on top of head.
34	*Paederia foetida* L. MPCW-UODA 3085/2013	Rubiaceae (Perennial)	Padri gota (Skunk vine)	Leaf	Diarrhea. Leaves are boiled in water followed by drinking the water.
35	*Aegle marmelos* (L.) Corr. MPCW-UODA 3086/2013	Rutaceae (Perennial)	Bael (Bengal quince)	Leaf	Stomach disorders, watery stool, loss of appetite. Young leaves are chewed followed by drinking water for stomach disorders. Juice obtained from crushed leaves is orally taken for watery stool. Leaves are chewed to increase appetite.
36	*Santalum album* L. MPCW-UODA 3087/2013	Santalaceae (Perennial)	Shada chandan (East Indian sandalwood)	Wood	See *Phyllanthus emblica*.
37	*Scoparia dulcis* L. MPCW-UODA 3088/2013	Scrophulariaceae (Perennial)	Boner dhonia (Goatweed)	Whole plant	The Deb barma clan prepares a wine from fermenting the whole plant and drinks it.
38	*Smilax macrophylla* Roxb. MPCW-UODA 3089/2013	Smilacaceae (Perennial)	Kumira lota	Stem	Weakness, tremor in hands or legs. Crushed stems are mixed with water. The water is taken orally in the form of a sherbet.
39	*Physalis micrantha* Link MPCW-UODA 3092/2013	Solanaceae (Annual)	Bon morich, Pagla pata (Cape gooseberry)	Leaf	Infections between the fingers of legs from working long hours in water. Crushed leaves are applied to infections.
40	*Pouzolzia zeylanica* (L.) Benn. MPCW-UODA 3096/2013	Urticaceae (Perennial)	Lajon turi (Graceful pouzolzs bush)	Leaf	Skin infections, skin disorders. Crushed leaves are applied as poultice on the affected areas of skin.
41	*Clerodendrum viscosum* Vent. MPCW-UODA 3097/2013	Verbenaceae (Perennial)	Bhatir pata, Vite gach (Hill glory bower)	Leaf	Jaundice, helminthiasis. Juice obtained from crushed leaves is taken orally.
42	*Lantana camara* L. MPCW-UODA 3099/2013	Verbenaceae (Perennial)	Motmoti (Spanish flag)	Leaf	Flatulence. Juice obtained from crushed leaves is taken orally.
43	*Alpinia nigra* (Gaertn.) Burtt. MPCW-UODA 3102/2013	Zingiberaceae (Annual)	Tara (Galingale)	Stem	Being possessed by ‘genies’ or ‘ghosts’. Stem is tied with a thread around the waist and worn in that position till the patient is ‘cured’.
44	*Curcuma longa* L. MPCW-UODA 3105/2013	Zingiberaceae (Perennial)	Holud (Turmeric)	Rhizome	See *Azadirachta indica*.

### Preferred mode of treatment by Deb barma clan households

Interview with all Heads of households and the adult persons of both sexes of the Deb barma clan suggested that in recent years, the clan is moving away from their traditional medicine towards treatment with allopathic medicine. In terms of household, 35% of households reported using only their traditional medicine and visiting their traditional medicinal healer, 20% reported visiting only allopathic doctors, 40% reported visiting both their traditional healer as well as the allopathic doctor, and 5% reported a combination of allopathic and homeopathic treatment. In terms of actual percent of persons using the various systems of medicine, 44.4% of the total clan population visited only their own clan healer, 14.8% visited only the allopathic doctor, 37.8% visited both their traditional clan healer as well as the allopathic doctor, and 3% of the total clan population received both allopathic and homeopathic treatments. The results are shown in Table 2. On further inquiries, the persons who visited the allopathic doctor only, mentioned that they have lost faith in their traditional healing methods, because allopathic treatment gave them quicker recoveries. People who visited both their clan healer as well as allopathic doctor mentioned that they visit their clan healer for simple diseases but go to allopathic doctor for treatment of life-threatening diseases. Sometimes, they visit their clan healer first, and if his treatment fails, they go to the allopathic doctor for treatment. People who visited both allopathic and homeopathic practitioners constituted a minority of the clan population. They visited the homeopathic physician for common diseases and also because homeopathic treatment was cheaper, and the allopathic physician for life-threatening diseases. When asked as to which diseases they thought to be common, it was the view point of most clan members that coughs and cold, or fever that goes away within a few days, or gastrointestinal disorders like flatulence were common diseases; most other diseases were regarded as complicated and which could become life-threatening. However, paranormal diseases like being possessed by ‘genies’ or ‘ghosts’ were always treated by their traditional healer. They also further mentioned that obtaining treatment from an allopathic doctor was a relatively new occurrence for them, and it happened only after a NGO operating in the area brought in the services of an allopathic doctor and advised the clan people to visit the doctor instead of their traditional healer.

**Table 2:** Preferred mode of treatment by Deb barma clan households

**Household Number**	**Total family member(s)**	**Preferred mode of treatment**			**Comments on preferred mode of treatment**
		**Traditional (Tribal healer)**	**Allopathic**	**Homeopathy**	
1	12	+	-	-	Tribal healer under all conditions.
2	6	+	+	-	Prefers allopathic over tribal healer.
3	5	+	+	-	Prefers allopathic for common diseases, tribal healer for paranormal diseases.
4	5	+	+	-	Prefers allopathic for common diseases, tribal healer for paranormal diseases.
5	7	-	+	-	Allopathic under all conditions.
6	10	+	-	-	Tribal healer under all conditions.
7	7	+	+	-	Prefers tribal healer for common diseases, allopathic for severe conditions.
8	8	+	+	-	Prefers tribal healer first, if treatment fails then goes for allopathic treatment.
9	12	+	-	-	Tribal healer under all conditions.
10	5	-	+	-	Allopathic under all conditions.
11	8	+	-	-	Tribal healer under all conditions.
12	4	-	+	+	Prefers allopathic for life-threatening diseases, homeopathy for common diseases.
13	7	+	-	-	Tribal healer under all conditions.
14	6	+	+	-	Prefers tribal healer for common diseases, allopathic for life-threatening diseases.
15	4	-	+	-	Allopathic under all conditions.
16	8	+	+	-	Prefers tribal healer first, allopathic only if tribal healer fails.
17	4	-	+	-	Allopathic under all conditions.
18	10	+	-	-	Tribal healer under all conditions.
19	1	+	-	-	Tribal healer under all conditions.
20	6	+	+	-	Prefers tribal healer for common diseases, allopathic for life-threatening diseases.

## Discussion

A number of the plants used by the Deb barma healer have been scientifically studied, or their use in traditional medicinal systems, particularly Ayurveda, has been described. Ayurveda is possibly the most ancient form of highly organized traditional medicinal system in the Indian sub-continent and dates back to almost 5,000 years ago. It is very much possible that since the Tripura tribe possibly came to India at least 2,000 years ago, there have been mutual interactions between the Ayurvedic medicinal system and the tribal medicines of the Tripura tribe including the Deb barma clan.

### Plants used in malaria

The Deb barma tribal healer used the plant, *Andrographis paniculata*, for treatment of malaria, a disease characterized by high fever. The plant is known in Ayurveda as ‘Kaalmegha’ and is used as a febrifuge, i.e. a medication that reduces fever [15]. Notably, in scientific studies, extract of this plant has been shown to possess anti-malarial activity through growth inhibition of *Plasmodium falciparum*, the parasite causing malaria [16]. However, the other plant used by the healer, *Justicia gendarussa*, to treat malaria, does not have any scientifically reported anti-malarial activity, and so is a promising plant for anti-malarial studies. On the other hand, *Justicia gendarussa* is known in Ayurveda as ‘Krishna Vaasaa’ and is used in the Ayurvedic formulary as a febrifuge.

### Plants used in skin diseases, tuberculosis and helminthiasis

*Justicia adhatoda* was used by the healer to treat skin infections and tuberculosis. Ethnomedicinal uses of the plant for treatment of tuberculosis have been reported from India [17]; in Ayurveda, the plant is known as ‘Vaasaka’ and is used as expectorant, and for bronchial and pulmonary afflictions, which would include tuberculosis [15]. Leaves of *Azadirachta indica* were used by the healer to treat ringworm infections, while bark was used to treat helmintic infections. In Ayurveda, the tree is known as ‘Nimba’, and the leaves and bark are considered anthelmintic and useful for treating skin infections. Feeding leaves, seeds or bark to small ruminants has also been shown to get rid of helminths from the ruminants [18]. Extract of leaves of the plant has also been reported to be effective against ringworm infections [19]. *Senna alata* leaves were used by the healer to treat eczema. In Ayurveda, the plant is known as ‘Dadrughna’ and is used to treat eczema [15]. Eczema is a disorder of the skin, and management of superficial skin infections with the use of soap containing *Senna alata* leaves have been reported [20].

### Plants used in pain or some diseases causing pain

*Crinum latifolium* was used by the healer to treat chest pain. Many *Crinum* species are in use worldwide in traditional medicinal systems for their analgesic properties [21]. *Colocasia esculenta*, used by the healer to treat rheumatic pain is known in Ayurveda as ‘Pindaaluka’ and is used in Ayurveda and other traditional medicinal systems of India for treatment of arthritis [22]. *Psidium guajava* leaf, used by the healer for treatment of tooth infections, has been shown to have beneficial effects on tooth ache [23], which usually accompanies tooth infections. The analgesic activity of *Plumbago indica* has also been reported [24], a plant used by the healer for treatment of pain. That the Deb barma healer possessed a good knowledge of the medicinal properties of plants is also evidenced by his use, respectively, of *Jatropha curcas* leaves and *Phyllanthus emblica* fruits for treatment of tooth ache and headache. Scientific studies have shown that the leaf extract of *Jatropha curcas* possess analgesic property [25,26]. Analgesic and anti-pyretic activity has also been reported for *Phyllanthus emblica* fruits [27].

### Plants used in gastrointestinal disorders

*Centella asiatica* was used by the healer to treat stomach disorders; the plant is also used in Ayurveda for treatment of gastrointestinal disorders, where the plant is known as ‘Manduukaparni’. The tribals of Meghalaya State in northeast India use the whole plant for treatment of diarrhea [28]. The Deb barma healer used the plant, *Ageratum conyzoides*, for treatment of stomach disorders. Use of this plant in traditional medicine for treatment of diarrhea has been reported from Nigeria [29].

Fruits of *Phyllanthus emblica* were used by the healer to increase appetite. In Ayurveda, the plant is known as ‘Aaamalaki’, and the fruits have multiple uses including that of being carminative, anti-diarrheal and as a gastrointestinal tonic [15]. The fruits of *Terminalia bellirica* were used along with fruits of *Terminalia chebula* and *Phyllanthus emblica* by the healer to increase appetite. *Terminalia bellirica* is known in Ayurveda as ‘Bibhitaka’ and its fruits are used for treatment of dyspepsia. *Terminalia chebula* is also considered an Ayurvedic plant (known in Ayurveda as ‘Haritaki’) and its fruits are used for treatment of flatulence and digestive disorders.

The stems of *Phyllanthus reticulatus* were used by the healer for treatment of diarrhea in children. In Ayurveda, the plant is known as ‘Kaamboji’, and the leaves are considered anti-diarrheal. Leaves of the plant (and possibly stems) are reported to contain lupeol [30]; the anti-diarrheal property of lupeol has been reported [31]. Thus the anti-diarrheal use of this plant by the Deb barma healer is in common with other traditional medicinal (Ayurveda) uses of the plant as well as scientifically validated.

*Aegle marmelos*, used by the healer for treating stomach disorders, is known in Ayurveda as ‘Bilva”, and considered a very specific plant for treatment of stomach complaints. *Paederia foetida*, also used by the healer for treatment of diarrhea, is known in Ayurveda as ‘Talanili’, and is considered an anti-diarrheal plant in this traditional medicinal system [15]. Leaves of *Lantana camara*, used by the healer to treat flatulence, are used by the Malayali tribals of Chitteri Hills in India to improve digestion in children [32]. It may be noted that flatulence can be caused because of indigestion.

### Plants used in coughs

Fruits of *Garcinia cowa* have been reported to be used in traditional medicines of Thailand for treatment of coughs [33], which use was similar to the use by the Deb barma healer. The flowers of *Leucas aspera* were used by the healer for treatment of coughs in infants. In Ayurveda, the plant is known as ‘Dronpushpi’, and the flowers are used to treat coughs and colds in children. The leaves of *Ocimum gratissimum* were used by the healer to treat rheumatic pain as well as coughs and mucus. Ayurvedic texts describe the plant as ‘Vriddha Tulasi’ and its uses for neurological and rheumatic afflictions [15]; scientific studies have validated the use of leaves of the plant for treatment of pain [34]; in homeopathy, the leaves are used to treat coughs.

### Plants used in diabetes and cardiovascular disorders

Bark of *Terminalia arjuna* was used by the healer to treat chest pain due to heart disorders; the aqueous extract of the bark has been shown to exert a cardiotonic effect on adult ventricular myocytes [35]. The therapeutic potential of bark of this plant in cardiovascular disorders has been reviewed [36]. It has further been shown that administration of bark extract of the plant improved myocardial function in streptozotocin-induced diabetic rats [37]; it is to be noted that diabetes can cause cardiovascular complications following onset of this disease. The plant is known in Ayurveda as ‘Arjuna’ and is used in Ayurvedic medicines as a cardioprotective and cardiotonic in angina and poor coronary circulation.

Leaves of *Lawsonia inermis* were used by the healer to treat diabetes; hypoglycemic activity of leaf extract has been reported in alloxan-induced diabetic mice [38]. The use of another plant by the Deb barma healer has been scientifically validated. Fruits of *Ficus hispida* were used by the healer to treat diabetes; bark extract of the plant has been shown to demonstrate hypoglycemic activity in normal and diabetic albino rats [39].

### Plants used in jaundice and other ailments

Leaves of *Moringa oleifera*, used by the healer to treat jaundice, have been shown to have hepatoprotective effect [40]. The hepatoprotective property of *Clerodendrum viscosum* has been shown [41], a plant used by the healer for treatment of jaundice. The leaves of *Melastoma malabathricum* were used by the healer to stop bleeding from external cuts and wounds; in some parts of India, the bark is also used for the same purpose [15].

### Comparative analysis of Deb barma tribal use of medicinal plants with other reported tribal uses in Bangladesh

We have previously conducted ethnomedicinal surveys of the Harbang clan of the Tripura tribe [12], who inhabits the southeastern portion of Bangladesh, as well as Tripura tribal communities residing in other parts of the country. The present survey was conducted on the Deb barma clan of the same tribe, who inhabits the northeastern part of Bangladesh. It is of interest that the medicinal plants used by the two clan healers (Harbang and Deb barma), with the exception of a few plants, were totally different. Even when the plants used were the same, the ailments treated were different. For instance, *Justicia adhatoda* was used by the Deb barma healer to treat skin infections and tuberculosis, but used by the Harbang clan healer to treat coughs and asthma. *Ageratum conyzoides* was used by the Deb barma healer to treat stomach disorders, but used by the Harbang healer to treat asthma. However, *Terminalia arjuna* was used by both clan healers for treatment of heart disorders. It is possible that the two clans being separated into two regions used different medicinal plants more available in their vicinity for treatment. It is also possible that the choice of Deb barma medicinal plants were influenced by interactions with Ayurvedic practitioners, while the Harbang clan selection of medicinal plants reflects choices of a more indigenous nature, i.e. influenced by experiences of their own tribal healer. More studies need to be conducted in this regard on possible interactions of Ayurveda with medicines of various Tripura tribal clans.

Our previous studies on various tribes point to both similarities and differences between medicinal uses of plants between the Deb barma clan and other tribes, with differences being more than similarities. For instance, the Santal tribe of Rangpur district, Bangladesh use whole plants of *Colocasia esculenta* for treatment of diarrhea, dysentery, piles, and wounds [4]; the Deb barma healer used tubers of the plant for treatment of rheumatic pain. The Hodi tribe uses the same plant for treatment of prolapse of uterus [6]. The plant is used for treatment of stomach pain and hiccup by the Tripura community of Hazarikhil in Chittagong district [42]; and for treatment of diabetes by the Teli clan of the Telegu tribe [43]. *Ageratum conyzoides* was used by the Santal healer for treatment of impotency [4], but by the Deb barma healer against stomach disorders. The plant was used as an insect repellent and for treatment of wounds and itches by the Garo tribe inhabiting Netrakona district [44]; and for treatment of bleeding, acidity, stomach pain by the Marma tribal community residing in Naikhongchaari, Bandarban district [45]. The plant was also used for treatment of bleeding from cuts and wounds by the Naik clan of the Rajbongshi tribe of Moulvibazar district [46]; and for treatment of severe headache by the Sigibe clan of the Khumi tribe residing in Thanchi sub-district of Bandarban district [47]. Thus, in this case, the Deb barma use was the same as the Marma tribal use in the sense that both tribes used the plant for stomach disorders but differed from the rest of the tribes surveyed.

The Santal healer used *Moringa oleifera* against constipation, epilepsy, skin eruptions, leucoderma, and as an astringent [4], while the Deb barma healer used the plant against jaundice. The Pahan tribe uses the plant against rheumatism, chicken pox, and as snake repellent [8]. The Sardar community used seeds and fruits of *Terminalia bellirica* against osteoporosis, diabetes, hysteria, cardiovascular disorders, and low density of semen and kidney problems [7], but the Deb barma healer used fruits of the plant to improve appetite. However, the fruits are used also to treat long-term fever, loss of appetite and as a sexual stimulant by the healers of Tripura tribe residing in Chittagong Hill Tracts [48]. In this case, regarding treatment of loss of appetite (or to improve appetite), the Tripura tribal use of fruits of *Terminalia bellirica* was the same between the Deb barma clan of the Tripura tribe (residing in Moulvibazar district in the northeast part of Bangladesh) and the Tripura tribal community residing in Chittagong Hill tracts (in the southeast part of Bangladesh). Notably, the plant and especially the fruits are also used as aphrodisiac, energizer, and for treatment of fever, and body ache by the Tonchongya tribal community of Roangchaari sub-district of Bandarban district [49]; treatment of urinary tract infection, hysteria by Tripura community of Hazarikhil in Chittagong district [42] (differences in the plant use by this Tripura community with the other Tripura clans and communities to be noted); treatment of anemia by the Pankho community of Bilaichari Union in Rangamati district [50]; treatment of coughs by the Kanda tribe of Sylhet district [51]; and treatment of coughs and diarrhea by the Rakhaing community of Cox’s Bazar district [52].

The Rai tribe uses *Paederia foetida* against insanity and mental disorders [10], but the Deb barma healer used the plant against diarrhea. The plant is used for treatment of rheumatic pain, burning sensations during urination by the healers (tribal medicinal practitioners or TMPs) of the Baburo, Haduga and Larma clans of the Chakma tribe residing in Rangamati district [53], and for treatment of toothache by the Bongshi tribe of Tangail district [54]. Taken together, the findings indicate that although certain therapeutic uses of the same plant may be similar, a higher degree of differences exist between medicinal uses of the same plant and its various parts between the various tribes of Bangladesh, which underscores the necessity of documenting medicinal practices of as many tribes as possible to get a comprehensive picture of the manifold uses of any given plant species.

### Review of ethnomedicinal uses of Deb barma plants with other reported folk medicinal uses in Bangladesh

A review of the various reported ethnomedicinal uses in Bangladesh of the plants of the Deb barma healer is shown in Table 3. The Bangladeshi traditional medicine has been described as a “unique conglomerate of different ethnomedicinal influences” [55]. Besides the more widely known Ayurveda and Unani systems of medicine with their established colleges and pharmacopoeias, folk and tribal medicinal systems, respectively, play an important role in providing health care services to the mainstream particularly rural Bengali-speaking population and the tribal people. To some extent, some of our surveys [56] as well as Table 3 indicate that these medicinal systems influence each other (more so with Ayurvedic medicine influencing folk and tribal medicines as well as quite possibly the other way round). Folk and tribal medicinal practitioners have several things in common; primarily they rely on simple formulations of medicinal plants for treatment with occasional uses of animal parts, incantations and amulets. Also the medicinal formulations are to a great extent highly individualistic in the sense that formulations can vary greatly from practitioner to practitioner, even though the practitioners may be practicing in the same village or adjoining villages [57-60]. This can be also seen in Table 3, where healers from various areas of Bangladesh can be seen to use the listed plants in a highly diversified manner.

**Table 3:** Other reported ethnomedicinal uses in Bangladesh of medicinal plants of the Deb barma healer

**Plant name**	**Reported ethnomedicinal uses in Bangladesh**
*Andrographis paniculata* Nees	Lung infections, liver disorders by the Teli tribe [8]; helminthiasis, fever by the Kole tribe [10]; fever and malarial fever by the Bauri tribe [61]; fever, headache, vertigo by the Garo tribe inhabiting Madhupur forest region [62]; intestinal worms, low sperm count, jaundice, skin disorders, liver dysfunction by the folk medicinal practitioners (FMPs) of Jessore district [63]; liver diseases by FMPs of Feni district [64]; stomach and heart disorders by the Goala tribe [65]; indigestion by FMPs of Rampal in Bagerhat district [57]; anorexia by FMPs of Rampal in Bagerhat district [58]; diabetes in Dhaka [55]; liver diseases, helminthiasis by FMPs of Shitol Para village, Jhalokati district [66]; emetic, helminthiasis, sexual disorders by FMPs of villages in Natore and Rajshahi districts [67]; helminthiasis, dysentery, rectal diseases, coughs, cold, fever, mucus by FMPs of Bheramara area in Kushtia district [68]; fever by FMPs of villages by the Padma River in Rajshahi district [59]; fever, intestinal and hepatic disorders by FMPs of five villages in Narsinghdi district [69]; liver disorders, helminthiasis, acidity by FMPs of Vitbilia village of Pabna district [70]; fever, intestinal and hepatic disorders by FMPs of Paschim Shawra and Palordi villages of Gaurnadi sub-district in Barisal district [71]; edema, blood purifier, to strengthen stomach and liver functions, helminthiasis, debility, loss of appetite, fever, skin infections by FMPs of Khulna City, Bangladesh [72]; diabetes, leucorrhea by FMPs of Fulbaria, Baguri, and Bagh-achra villages in Jessore district [73]; helminthiasis in children by FMPs of Barobazar village, Jhenidaha district [74]; headache by FMPs of Jool chotro and Janga lia villages of Tangail district [75]; fever by FMPs of six villages in Thakurgaon district [76]; fever, constipation by the Bongshi tribe of Tangail district [54]; stomach disorders, to improve digestion, bloating with burning sensations in the chest, by FMPs of several villages of Faridpur and Rajbari districts [77]; stomach and heart disorders by the Goala tribe of Moulvibazar district [65]; diabetes by the Soren clan of the Santal tribe residing in Nobogram village in Rajshahi district [78]; fever, intestinal worm, spleenomegally by Tripura community of Hazarikhil in Chittagong district [42]; common cold, uncomplicated sinusitis, pharyngotonsillitis, lower urinary tract infections, acute diarrhea by FMPs of Shat-tola Bazaar and Talbari villages in Bagerhat district [79]; cold, coughs, fever by the Khatriya and Kashya clans of the Bagdi tribe of Rajbari district [80]; fever arising suddenly during the night, skin infections, toothache by the Tripura tribe residing in Comilla district [81].
*Justicia adhatoda* L.	Coughs, fever by the Teli tribe [8]; coughs, mucus, asthma by the Harbang clan of the Tripura tribe [12]; coughs by the Bauri tribe [61]; pain, cold, coughs, asthma, wounds by FMPs of Noakhali district [64]; tuberculosis by FMPs in Tangail district [82]; coughs, pneumonia, asthma by the Garo tribe inhabiting Netrakona district [44]; respiratory problems by FMPs of Sylhet Division [83]; asthma by the Soren clan of the Santal tribe residing in Kannapara and Mondumala villages of Rajshahi district [84]; helminthiasis, diarrhea, constipation by the Marma tribal community residing in Naikhongchaari, Bandarban district [45]; coughs, asthma, bleeding hemorrhoids by FMPs of Shitol Para village, Jhalokati district [66]; helminthiasis, coughs, sedative, sprain by FMPs of villages in Natore and Rajshahi districts [67]; coughs by FMPs of Daudkandi sub-district in Comilla district [85]; coughs, body ache by FMPs and tribal medicinal practitioners (TMPs) of Khakiachora village in Sylhet district [86]; mucus by FMPs of Dhamrai sub-district of Dhaka district [87]; rabies, pneumonia, jaundice by FMPs of Rahmatpur village by the Ghaghot River, Rangpur district [59]; antidote to poisoning, bronchitis, malaria, skin eruptions, astringent, cold in cattle by FMPs of villages by the Bangali River in Bogra district [59]; fever, cold, coughs by FMPs of Balidha village in Jessore district [88]; coughs, asthma, menstrual problems, jaundice, hepatitis by FMPs of Station Purbo Para village in Jamalpur district [89]; asthma by FMPs of Shetabganj village in Dinajpur district [90]; coughs, asthma by FMPs of Shekhertek and Badarganj villages in Rangpur district [91]; leucorrhea, chronic respiratory disorders, coughs by FMPs of Daulatdia Ghat in Kushtia district [92]; coughs by FMPs of Barisal Town in Barisal district [93] and by FMPs of three areas of Pirojpur district [94]; all types of pain, fever by FMPs of three villages in Panchagarh and Thakurgaon districts [95]; whooping cough by FMPs of Vitbilia village of Pabna district [70]; severe fever with mucus by FMPs of six villages in Greater Naogaon district [96]; chronic asthma, leprosy by FMPs of seven villages of Ishwardy sub-district in Pabna district [97]; tuberculosis by FMPs of Babla and Terbaria villages in Tangail district [82]; coughs, biliary problems, frequent thirsts, fever, vomiting tendency, diabetes, leprosy, tuberculosis by FMPs of three villages in Sreepur sub-district in Magura district [98]; upper respiratory infections, chest pain with coughs, bronchitis, long-term fever with abnormally high body temperatures by FMPs of Paschim Shawra and Palordi villages of Gaurnadi sub-district in Barisal district [71]; coughs, blood coming ou with coughs, fever, mucus, asthma, all sorts of pain including pain and swelling due to injury, bronchitis, respiratory difficulties by FMPs of Uttar Musrat Madati and Kisasat Madati villages in Lalmonirhat district [60]; tuberculosis, blood purifier, spleen disorders, burning sensations during urination, antidote to poisoning, coughs, whooping coughs, mucus, asthma, fever, jaundice, vomiting or vomiting tendency, gonorrhea, leprosy, to regularize menstruation, stoppage of urination by FMPs of Khulna City, Bangladesh [72]; whooping cough by FMPs of Fulbaria, Baguri, and Bagh-achra villages in Jessore district [73]; frequent fever by FMPs of Shonapur, Chorkulte, and Majhbari villages of Rajbari district [99]; coughs, whooping cough, fever, pneumonia by FMPs of Arpara and Munshefpur villages in Jessore district [100]; chronic cough, asthma by FMPs of Barobazar village, Jhenidaha district [74]; asthma, jaundice by FMPs of Jool chotro and Janga lia villages of Tangail district [75]; cold, fever, jaundice by FMPs of Jool chotro and Janga lia villages of Tangail district [75]; cold, fever, jaundice by FMPs of six villages in Thakurgaon district [76]; respiratory difficulties, asthma by the FMP of Kasipur village in Narayanganj district [101]; coughs, mucus by FMPs of Baghbhandar, Sonahat and Kumarpara villages in Kurigram district [102]; coughs by the Naik clan of the Rajbongshi tribe of Moulvibazar district [46]; malaria, coughs, cold by the Tonchongya tribal community of Roangchaari sub-district of Bandarban district [49]; coughs by Tripura community of Hazarikhil in Chittagong district [42]; coughs, chest pain, pneumonia by the Hajong community of Baromari village in Netrakona district [103]; cold, coughs by FMPs of four villages in Natore and Rajshahi districts [104]; coughs by the Pankho community of Bilaichari Union in Rangamati district [50]; skin infections, skin diseases by the Kanda tribe of Sylhet district [51]; coughs by the Rakhaing community of Cox’s Bazar district [52]; flatulency, low sperm count, astringent, biliary problems, diarrhea, dysentery, tuberculosis, coughs, fever, eczema, leprosy, asthma by FMPs of Shat-tola Bazaar and Talbari villages in Bagerhat district [79]; coughs, tuberculosis by the Tripura tribe residing in Comilla district [81]; coughs, mucus, fever, tuberculosis by folk medicinal herbalists in seven villages of Bhola district [105]; coughs, mucus by the Teli clan of the Telegu tribe [43]; chest pain, coughs, cold, asthma by the Chakma community of Chittagong Hill Tracts [106].
*Justicia gendarussa* Burm. f.	Infertility with seizures by the Hodi tribe [6]; insect bite, nocturnal emissions by the Khasia tribe [11]; fever, coughs, mucus, asthma, whitish discharge in urine of men or women by the Bauri tribe [61]; wounds by the Garo tribe inhabiting Madhupur forest region [62]; eczema, dysentery, jaundice, rheumatism, animal or bird bites by FMPs of Noakhali district [64]; bone fracture and fracture-associated pain by the Garo tribe inhabiting Netrakona district [44]; bone fracture, rheumatic pain by FMPs of Sylhet Division [83]; back pain by FMPs of a group of Christians residing in Mirzapore village of Dinajpur district [107]; appetizer, helminthiasis by FMPs of villages in Natore and Rajshahi districts [67]; jaundice by FMPs of Rahmatpur village by the Ghaghot River, Rangpur district [59]; debility by FMPs of Vasu Bihar village, Bogra district [108]; cuts and wounds, substitute for anti-tetanus injection by FMPs of Station Purbo Para village in Jamalpur district [89]; rheumatic pain by FMPs of Shetabganj village in Dinajpur district [90]; flatulence in cattle by FMPs of three areas of Pirojpur district [94]; wasting away of body by FMPs of three villages in Panchagarh and Thakurgaon districts [95]; paralysis by FMPs of six villages in Greater Naogaon district [96]; bleeding from external cuts and wounds, bleeding through the mouth, excessive or irregular bleeding during menstruation by FMPs of Uttar Musrat Madati and Kisasat Madati villages in Lalmonirhat district [60]; snake bite by FMPs of Shonapur, Chorkulte, and Majhbari villages of Rajbari district [99]; rheumatic pain by the Bongshi tribe of Tangail district [54]; rheumatic pain by FMPs of several villages of Faridpur and Rajbari districts [77]; coughs and mucus in children by the Naik clan of the Rajbongshi tribe of Moulvibazar district [46]; paralysis by the Rai Kshatriya tribe of Pabna district [109]; throat pain due to cold and coughs by the Sigibe clan of the Khumi tribe residing in Thanchi sub-district of Bandarban district [47]; liver disorder, astringent by the Tonchongya tribal community of Roangchaari sub-district of Bandarban district [49]; fever, cold, ear lobe infection by FMPs of four villages in Natore and Rajshahi districts [104]; helmintic infections in children by the Kanda tribe of Sylhet district [51]; to bring out poison by the Khatriya and Kashya clans of the Bagdi tribe of Rajbari district [80].
*Aerva sanguinolenta* (L.) Blume	Bleeding from cuts and wounds by the Bauri tribe [61]; rheumatism, leucorrhea by FMPs of Noakhali district [64]; tonic, sedative, dermatitis by FMPs of villages in Natore and Rajshahi districts [67]; bleeding from cuts and wounds by FMPs and tribal medicinal practitioners (TMPs) of Khakiachora village in Sylhet district [86]; blood in urine by the ojhas (tribal medicinal practitioners) of the Santal tribe of Rajshahi district [110]; bone fracture, abortifacient by FMPs of Rahmatpur village by the Ghaghot River, Rangpur district [59]; body pain by FMPs of Vitbilia village of Pabna district [70]; malnutrition in newly delivered mother by FMPs of Uttar Musrat Madati and Kisasat Madati villages in Lalmonirhat district [60]; snake bite, stomach disorder by FMPs of Shonapur, Chorkulte, and Majhbari villages of Rajbari district [99]; bleeding from cuts and wounds by the Bongshi tribe of Tangail district [54]; insect sting, allergic rash by FMPs of four villages in Natore and Rajshahi districts [104]; insect or snake bite by the Pankho community of Bilaichari Union in Rangamati district [50].
*Crinum latifolium* L.	Indigestion in cattle by the Khasia tribe [11]; indigestion by the Santal tribe residing in Thakurgaon district [111]; antidote to poisoning, skin disorder, cattle sedative, conjunctivitis in cattle by FMPs of villages by the Bangali River in Bogra district [59].
*Centella asiatica* (L.) Urb.	Jaundice by the Hodi tribe [6]; helminthiasis, stomach ache by the Harbang clan of the Tripura tribe [12]; dysentery by the Bauri tribe [61]; indigestion, stomach infection by the Garo tribe inhabiting Madhupur forest region [62]; weakness, skin problems, dysentery, indigestion, cataract, gonorrhea, low semen density, leucorrhea by FMPs of Noakhali district [64]; fever, pain by FMPs in Tangail district [82]; dysentery, intestinal pain by the Garo tribe inhabiting Netrakona district [44]; indigestion, appetite stimulant by FMPs of Sylhet Division [83]; dysentery, stomach ache, to increase memory by FMPs of Shitol Para village, Jhalokati district [66]; body ache, dysentery by FMPs of Daudkandi sub-district in Comilla district [85]; gastric or liver troubles by FMPs and tribal medicinal practitioners (TMPs) of Khakiachora village in Sylhet district [86]; diarrhea, gastric problems by FMPs of Dhamrai sub-district of Dhaka district [87]; gastric disorder, stomach pain, diarrhea, blood dysentery, fever, coughs by the TMPs of Tripura tribe residing in Chittagong Hill Tracts [48]; bone fracture by FMPs of Rahmatpur village by the Ghaghot River, Rangpur district [59]; sexual diseases, uncontrolled urination by FMPs of villages by the Padma River in Rajshahi district [59]; cold, rabies, gastric ulcer, dysentery, intestinal disorders by FMPs of five villages in Narsinghdi district [69]; dysentery by FMPs of Vasu Bihar village, Bogra district [108]; dysentery, cataract, stomach problems by FMPs of Balidha village in Jessore district [88]; cataract in goats, to keep head cool, diabetes, swelling in eyes, conjunctivitis in humans by FMPs of Station Purbo Para village in Jamalpur district [89]; hair loss, dysentery, gastrointestinal disorders by FMPs of Shetabganj village in Dinajpur district [90]; gastrointestinal disorders by FMPs of Shekhertek and Badarganj villages in Rangpur district [91]; blood purifier, fever, diabetes by FMPs of Daulatdia Ghat in Kushtia district [92]; ulcer by FMPs of three areas of Pirojpur district [94]; anemia, vomiting, stomach pain by FMPs of six villages in Greater Naogaon district [96]; cold, dysentery, blood purifier by FMPs of seven villages of Ishwardy sub-district in Pabna district [97]; fever, pain by FMPs of Babla and Terbaria villages in Tangail district [82]; indigestion, flatulence, helminthiasis, diarrhea by FMPs of Paschim Shawra and Palordi villages of Gaurnadi sub-district in Barisal district [71]; blood dysentery by FMPs of Shonapur, Chorkulte, and Majhbari villages of Rajbari district [99]; stomach pain by FMPs of Arpara and Munshefpur villages in Jessore district [100]; cold, rabies, gastric problems, ulcer, dysentery by FMPs of six villages in Thakurgaon district [76]; diarrhea, dysentery by the FMP of Kasipur village in Narayanganj district [101]; flatulence, indigestion, hair loss by FMPs of Baghbhandar, Sonahat and Kumarpara villages in Kurigram district [102]; anemia by TMPs of a Mro tribal community residing at Gazalia Union of Bandarbans district [112]; jaundice, dysentery by FMPs of several villages of Faridpur and Rajbari districts [77]; stomach pain by the Naik clan of the Rajbongshi tribe of Moulvibazar district [46]; to increase memory by a FMP of Savar in Dhaka district [113]; stomach pain in children, dysentery by the Rai Kshatriya tribe of Pabna district [109]; pain, dysentery, diarrhea, flatulence, tuberculosis by Tripura community of Hazarikhil in Chittagong district [42]; fever, pyorrhea, impotency, gastritis, jaundice by the Rakhaing community of Cox’s Bazar district [52]; stomach pain, dysentery by the Tudu sub-clan of the Santal tribe in Joypurhat district [114]; gastrointestinal disorders, to increase memory by folk medicinal herbalists in seven villages of Bhola district [105]; diarrhea, dysentery by the Teli clan of the Telegu tribe [43]; syphilis, ulcer by the Chakma community of Chittagong Hill Tracts [106].
*Alstonia scholaris* (L.) R. Br.	Abscess, swelling of gums, asthma by the Bauri tribe [61]; leucorrhea by the Garo tribe inhabiting Madhupur forest region [62]; cancer sand tumors by FMPs of Rampal in Bagerhat district [58]; kidney and hepatic problems in humans, restlessness in cattle by FMPs in Tangail district [82]; nerve stimulant by FMPs of Sylhet Division [83]; cold sores (caused by herpes), fever, diabetes by the Marma tribal community residing in Naikhongchaari, Bandarban district [45]; gastric problems by FMPs of a group of Christians residing in Mirzapore village of Dinajpur district [107]; puerperal fever, pain, jaundice by FMPs and tribal medicinal practitioners (TMPs) of Khakiachora village in Sylhet district [86]; debility by the Santal tribe residing in Thakurgaon district [111]; syphilis, skin diseases, leprosy by FMPs of Rahmatpur village by the Ghaghot River, Rangpur district [59]; rheumatic pain, fever, dysentery by FMPs of five villages in Narsinghdi district [69]; infections accompanied by swellings on human face, ulcer by FMPs of Station Purbo Para village in Jamalpur district [89]; allergy by FMPs of Shetabganj village in Dinajpur district [90]; continuous fever, malaria by FMPs of Barisal Town in Barisal district [93]; to increase libido by FMPs of three villages in Panchagarh and Thakurgaon districts [95]; kala azar by FMPs of six villages in Greater Naogaon district [96]; fever, pimples, coughs, anti-bilious by by FMPs of seven villages of Ishwardy sub-district in Pabna district [97]; kidney and hepatic problems in humans, restlessness in cows by FMPs of Babla and Terbaria villages in Tangail district [82]; malaria by FMPs of Paschim Shawra and Palordi villages of Gaurnadi sub-district in Barisal district [71]; mouth ulcer by FMPs of six villages in Thakurgaon district [76]; galactagogue by the TMPs of the Baburo, Haduga and Larma clans of the Chakma tribe residing in Rangamati district [53]; aphrodisiac, antidote to poisoning, inflammation, fever by the Tonchongya tribal community of Roangchaari sub-district of Bandarban district [49]; lip blister by the Rakhaing community of Cox’s Bazar district [52]; acne, coughs, flatulence, blood disorder, asthma, abdominal tumor, chronic enlargement of spleen, helminthiasis by FMPs of Shat-tola Bazaar and Talbari villages in Bagerhat district [79]; jaundice by the Khatriya and Kashya clans of the Bagdi tribe of Rajbari district [80]; skin infections by the Tripura tribe residing in Comilla district [81]; skin infections, dysentery, diarrhea, fever by folk medicinal herbalists in seven villages of Bhola district [105].
*Colocasia esculenta* (L.) Schott.	Hemorrhoids, diarrhea, dysentery, wound by the Santal tribe [4]; prolapse of uterus by the Hodi tribe [6]; cuts and wounds by the Garo tribe inhabiting Netrakona district [44]; severe jaundice, digestive aid, constipation by FMPs of Shitol Para village, Jhalokati district [66]; colic, indigestion by FMPs of villages in Natore and Rajshahi districts [67]; anti-hemorrhagic, blood purifier, to strengthen bones by FMPs of Dhamrai sub-district of Dhaka district [87]; astringent, carminative, scar, tumor, infertility by FMPs of villages by the Bangali River in Bogra district [59]; allergic disorders by FMPs of five villages in Narsinghdi district [69]; astringent, bloating, dermatitis, tiger bite, helminthiasis, emetic by FMPs of two villages by the Rupsha River in Bagerhat district [115]; rheumatic pain, paralysis by FMPs of Baghbhandar, Sonahat and Kumarpara villages in Kurigram district [102]; stomach pain, hiccup by Tripura community of Hazarikhil in Chittagong district [42]; diabetes by the Teli clan of the Telegu tribe [43].
*Borassus flabellifer* L.	Jaundice by the Sardar community [7]; ear ache by the Kole tribe [10]; pain during menstruation by the Bauri tribe [61]; debility, insomnia by the Garo tribe inhabiting Netrakona district [44]; cancer, edema, epilepsy, boils by FMPs in five villages of Boalia sub-district, Rajshahi district [116]; low sperm count by FMPs and tribal medicinal practitioners (TMPs) of Khakiachora village in Sylhet district [86]; edema, inflammation by FMPs of three areas of Pirojpur district [94]; wasting away of body by FMPs of three villages in Panchagarh and Thakurgaon districts [95]; urinary problems arising from diabetes or endocrinological disorders by FMPs of six villages in Greater Naogaon district [96]; having difficulties in urinating, excessive bleeding following abortion by TMPs of a Mro tribal community residing at Gazalia Union of Bandarbans district [112]; sexual weakness by a FMP of Savar in Dhaka district [113].
*Asparagus racemosus* Willd.	Physical weakness by the Kole tribe [10]; bacterial or fungal infections, edema, tonic, bloating, hypertension, galactagogue, malnutrition in children, memory and strength enhancer, nerve weakness by FMPs of Noakhali district [64]; snake bite, wounds by the Garo tribe inhabiting Netrakona district [44]; filariasis, sexual dysfunction in men, night blindness by the Soren clan of the Santal tribe residing in Kannapara and Mondumala villages of Rajshahi district [84]; diabetes, tuberculosis by FMPs of Shitol Para village, Jhalokati district [66]; sexual disorders, anti-hemorrhagic, sore throat, night blindness, blood dysentery by FMPs of villages in Natore and Rajshahi districts [67]; stone dislodged in penis, diabetes by FMPs of Bheramara area in Kushtia district [68]; tuberculosis by the ojhas (tribal medicinal practitioners) of the Santal tribe of Rajshahi district [110]; burning sensations during urination, bloating by FMPs of Rahmatpur village by the Ghaghot River, Rangpur district [59]; diabetes, heart diseases by FMPs of Balidha village in Jessore district [88]; debility, to keep body healthy by FMPs of Station Purbo Para village in Jamalpur district [89]; physical weakness in males by FMPs of Shetabganj village in Dinajpur district [90]; constipation, impotency in men by FMPs of Vitbilia village of Pabna district [70]; to increase libido by FMPs of six villages in Greater Naogaon district [96]; hypertension, galactagogue by FMPs of seven villages of Ishwardy sub-district in Pabna district [97]; asthma (during winter), bitter taste in mouth, nutritive, to increase intelligence, to maintain good eyes, to increase sperm, to increase strength, galactagogue, diarrhea by FMPs of three villages in Sreepur sub-district in Magura district [98]; physical weakness by FMPs of Uttar Musrat Madati and Kisasat Madati villages in Lalmonirhat district [60]; mental, physical, nerve and sexual weakness, galactagogue, strengthen teeth, gonorrhea by FMPs of Khulna City, Bangladesh [72]; burning sensations in stomach, burning sensations in soles of feet, stomach pain, hot feeling in body, insomnia by FMPs of Shonapur, Chorkulte, and Majhbari villages of Rajbari district [99]; leucorrhea by FMPs of Arpara and Munshefpur villages in Jessore district [100]; leucorrhea, syphilis, gonorrhea by FMPs of Barobazar village, Jhenidaha district [74]; decreased sperm count, passing of semen with urine by FMPs of Jool chotro and Janga lia villages of Tangail district [75]; debility (in case of males) by FMPs of six villages in Thakurgaon district [76]; ‘meho’ (endocrinological disorders) by FMPs of Baghbhandar, Sonahat and Kumarpara villages in Kurigram district [102]; diabetes, debility by the Mandai tribe of Bangladesh [117]; asthma, leucorrhea by the Rai Kshatriya tribe of Pabna district [109]; asthma, coughs, cold by the Tonchongya tribal community of Roangchaari sub-district of Bandarban district [49]; vaginitis by the Chakma tribe of Hill Tracts districts [118]; labor problem, leucorrhea by Tripura community of Hazarikhil in Chittagong district [42]; weakness, diabetes, urinary problems by FMPs of four villages in Natore and Rajshahi districts [104]; swelling or enlargement of testicles by the Pankho community of Bilaichari Union in Rangamati district [50]; asthma during winter, bitter taste in mouth, nutritive, to increase intelligence, to maintain good eyes, to increase strength, to increase sperm, galactagogue, diarrhea, hyperacidity by FMPs of Shat-tola Bazaar and Talbari villages in Bagerhat district [79]; snake bite by the Khatriya and Kashya clans of the Bagdi tribe of Rajbari district [80].
*Ageratum conyzoides* L.	Astringent, impotency, insect repellent by the Santal tribe [4]; asthma by the Harbang clan of the Tripura tribe [12]; diarrhea, dysentery, boils, skin diseases, joint pain by FMPs of Noakhali district [64]; to expedite delivery in cows by FMPs in Tangail district [82]; insect repellent, wounds, itches by the Garo tribe inhabiting Netrakona district [44]; bleeding, acidity, stomach pain by the Marma tribal community residing in Naikhongchaari, Bandarban district [45]; insecticidal, coughs, fever, inflammation, spermatorrhea by FMPs of villages by the Bangali River in Bogra district [59]; to expedite delivery in cows by FMPs of Babla and Terbaria villages in Tangail district [82]; cuta and wounds, acne, skin diseases, fever by the FMP of Kasipur village in Narayanganj district [101]; bleeding from cuts and wounds by the Naik clan of the Rajbongshi tribe of Moulvibazar district [46]; severe headache by the Sigibe clan of the Khumi tribe residing in Thanchi sub-district of Bandarban district [47]; aphrodisiac by FMPs of four villages in Natore and Rajshahi districts [104]; coughs in infants by healers among tea garden workers in Sreemangal [119].
*Garcinia cowa* Roxb.	Dried, powdered fruit used in headache and dysentery [120].
*Terminalia arjuna* (Roxb. ex DC.) Wight & Arn.	Depression on both sides of the head and chest with appearance of yellowish color in palm of hand and eyes by the Hodi tribe [6]; osteoporosis by the Sardar community [7]; heart disorders by the Harbang clan of the Tripura tribe [12]; cardiovascular disorders, whitish discharge during urination, burning sensations during urination, puerperal fever by the Bauri tribe [61]; low sperm count, diabetes, heart diseases by the folk medicinal practitioners (FMPs) of Jessore district [63]; stomach and heart disorders, premature graying of hair by the Goala tribe [65]; diabetes in Dhaka [55]; heart disease, diarrhea, dysentery, jaundice by the Garo tribe inhabiting Netrakona district [44]; cardiovascular disorders, appetite stimulant by FMPs of Sylhet Division [83]; hypertension, anemia, leprosy by FMPs in five villages of Boalia sub-district, Rajshahi district [116]; cardiovascular diseases, cholera, bleeding hemorrhoids, blood dysentery by FMPs of Shitol Para village, Jhalokati district [66]; leucorrhea, rheumatoid arthritis, infertility, weakness by FMPs of villages in Natore and Rajshahi districts [67]; sex stimulant, heart disease by FMPs of Daudkandi sub-district in Comilla district [85]; pain in bones by FMPs and tribal medicinal practitioners (TMPs) of Khakiachora village in Sylhet district [86]; heart disorders, hepatic disorders, jaundice, maintenance of normal blood pressure by the Rakhain tribe of Chittagong Hill Tracts [121]; erectile dysfunction by FMPs of villages by the Padma River in Rajshahi district [59]; cardiovascular disorders, blood purification by FMPs of five villages in Narsinghdi district [69]; heart disorders, indigestion by FMPs of Balidha village in Jessore district [88]; to increase sexual power, coughs, asthma, heart disorders, dysentery by FMPs of Station Purbo Para village in Jamalpur district [89]; low sperm count, dysentery by FMPs of Shekhertek and Badarganj villages in Rangpur district [91]; heart diseases by FMPs of Daulatdia Ghat in Kushtia district [92]; abnormal rhythms of heart by FMPs of Barisal Town in Barisal district [93]; abnormal heart beat by FMPs of three areas of Pirojpur district [94]; heart disorders, debility by FMPs of three villages in Panchagarh and Thakurgaon districts [95]; respiratory problems, coughs, fever, debility, hypotension by FMPs of six villages in Greater Naogaon district [96]; heart disease, bone fracture by FMPs of seven villages of Ishwardy sub-district in Pabna district [97]; heart diseases, gynecological disorders, nerve stimulant, leprosy, gonorrhea by FMPs of two villages by the Rupsha River in Bagerhat district [115]; loss of sexual desire, heart disorders, constipation, infections from cuts and wounds, blood purifier, obesity, diabetes, coughs, acne by FMPs of three villages in Sreepur sub-district in Magura district [98]; paralysis by FMPs of four adjoining villages in Narail and Jessore districts [122]; heart disorders, chronic dysentery by FMPs of Uttar Musrat Madati and Kisasat Madati villages in Lalmonirhat district [60]; cardiovascular disorders by FMPs of Khulna City, Bangladesh [72]; cardiovascular disorders by FMPs of Fulbaria, Baguri, and Bagh-achra villages in Jessore district [73]; stomach disorders, cardiovascular disorders, diabetes by FMPs of Shonapur, Chorkulte, and Majhbari villages of Rajbari district [99]; decrease in libido by FMPs of Arpara and Munshefpur villages in Jessore district [100]; syphilis, gonorrhea, dysentery by FMPs of Barobazar village, Jhenidaha district [74]; weakness of heart, joint pain by FMPs of Jool chotro and Janga lia villages of Tangail district [75]; low sperm count, dysentery, heart disease by FMPs of six villages in Thakurgaon district [76]; heart disease, pain in heart, blood coming from mouth by FMPs of Baghbhandar, Sonahat and Kumarpara villages in Kurigram district [102]; spermatorrhea by the Bongshi tribe of Tangail district [54]; heart disorders, liver problem, hepatitis, kidney problem, passing of semen with urine by FMPs of several villages of Faridpur and Rajbari districts [77]; stomach and heart disorders, graying of hair by the Goala tribe of Moulvibazar district [65]; snake bite by the Rai Kshatriya tribe of Pabna district [109]; knee and waist pain by the Soren clan of the Santal tribe residing in Nobogram village in Rajshahi district [78]; scabies, itching by Tripura community of Hazarikhil in Chittagong district [42]; cardiovascular disorders by the Hajong community of Baromari village in Netrakona district [103]; scabies, itching by the Rakhaing community of Cox’s Bazar district [52]; dysentery, flatulency by the Murmu tribal community of Rajshahi district [123]; heart disorders by the Tripura tribe residing in Comilla district [81]; heart disorders, low semen density, coughs, blood purifier, leucorrhea by folk medicinal herbalists in seven villages of Bhola district [105]; blister by the Chakma community of Chittagong Hill Tracts [106].
*Terminalia bellirica* (Gaertn.) Roxb.	Diabetes, cardiovascular disorders, low density of semen, kidney problems, hysteria, osteoporosis by the Sardar community [7]; coughs, mucus, asthma by the Harbang clan of the Tripura tribe [12]; fever with shivering by the Bauri tribe [61]; tonic, diarrhea, dysentery, coughs, breathing problems, hair tonic, joint pain by FMPs of Noakhali district [64]; constipation by the Goala tribe [65]; diabetes in Dhaka [55]; coughs, to increase strength, appetite stimulant, to increase eye sight by FMPs of Sylhet Division [83]; constipation, sexual diseases by FMPs in five villages of Boalia sub-district, Rajshahi district [116]; stimulant, impotency by FMPs of villages in Natore and Rajshahi districts [67]; waist pain by FMPs and tribal medicinal practitioners (TMPs) of Khakiachora village in Sylhet district [86]; erectile dysfunction by FMPs of Bheramara area in Kushtia district [68]; long-term fever, loss of appetite, sexual stimulant by the TMPs of Tripura tribe residing in Chittagong Hill Tracts [48]; asthma, to maintain heart, lungs and liver in good condition by FMPs of Station Purbo Para village in Jamalpur district [89]; to increase libido, acidity by FMPs of Shekhertek and Badarganj villages in Rangpur district [91]; to keep healthy by FMPs of three areas of Pirojpur district [94]; gonorrhea by FMPs of three villages in Panchagarh and Thakurgaon districts [95]; to cure any disease by FMPs of Vitbilia village of Pabna district [70]; blood purifier, appetite stimulant by FMPs of six villages in Greater Naogaon district [96]; helminthiasis, loss of hair by FMPs of seven villages of Ishwardy sub-district in Pabna district [97]; astringent, coughs, biliary disorders, to maintain good eyes and hair, helminthiasis, breaking down of voice, thirst, vomiting tendency, rheumatism by FMPs of three villages in Sreepur sub-district in Magura district [98]; jaundice, to ensure proper bowel movement, any type of gastrointestinal disorders by FMPs of Uttar Musrat Madati and Kisasat Madati villages in Lalmonirhat district [60]; chronic dysentery, hemorrhoids, to blacken hair and strengthen hair roots, to increase sperm, conjunctivitis, emetic, fever due to biliary trouble, headache, diarrhea by FMPs of Khulna City, Bangladesh [72]; fever by FMPs of Arpara and Munshefpur villages in Jessore district [100]; syphilis, gonorrhea by FMPs of Barobazar village, Jhenidaha district [74]; dysentery, cholera, gastric problems by FMPs of Jool chotro and Janga lia villages of Tangail district [75]; coughs, indigestion by FMPs of six villages in Thakurgaon district [76]; abscess, burning sensations on skin, hemorrhoids by the TMPs of the Baburo, Haduga and Larma clans of the Chakma tribe residing in Rangamati district [53]; coughs, spleen disorders, gastrointestinal disorders by FMPs of Baghbhandar, Sonahat and Kumarpara villages in Kurigram district [102]; gastric problem by FMPs of several villages of Faridpur and Rajbari districts [77]; constipation by the Goala tribe of Moulvibazar district [65]; aphrodisiac, energizer, fever, body ache by the Tonchongya tribal community of Roangchaari sub-district of Bandarban district [49]; urinary tract infection, hysteria by Tripura community of Hazarikhil in Chittagong district [42]; anemia by the Pankho community of Bilaichari Union in Rangamati district [50]; coughs by the Kanda tribe of Sylhet district [51]; coughs, diarrhea by the Rakhaing community of Cox’s Bazar district [52]; blood purifier by healers among tea garden workers in Sreemangal [119]; breathing problem by the Chakma community of Chittagong Hill Tracts [106].
*Terminalia chebula* Retz.	Cardiovascular disorders, hysteria, osteoporosis by the Sardar community [7]; diabetes by the Marakh sect of the Garo tribe [9]; fever with shivering by the Bauri tribe [61]; stomachic by the Garo tribe inhabiting Madhupur forest region [62]; diabetes in Dhaka [55]; digestive, quenching thirst, blood dysentery, bloating, constipation by FMPs of Sylhet Division [83]; asthma, heart disorders, eye disorders, night blindness, itches by FMPs in five villages of Boalia sub-district, Rajshahi district [116]; constipation, nausea by FMPs of Shitol Para village, Jhalokati district [66]; infections, indigestion by FMPs of villages in Natore and Rajshahi districts [67]; purgative, cough relief by FMPs of Daudkandi sub-district in Comilla district [85]; waist pain by FMPs and tribal medicinal practitioners (TMPs) of Khakiachora village in Sylhet district [86]; erectile dysfunction by FMPs of Bheramara area in Kushtia district [68]; jaundice by FMPs of Rahmatpur village by the Ghaghot River, Rangpur district [59]; gastrointestinal disorders, heart disorders, debility, helminthiasis by FMPs of Station Purbo Para village in Jamalpur district [89]; constipation, less urination by FMPs of Daulatdia Ghat in Kushtia district [92]; loss of sensitivity in skin due to allergy or other reasons, chronic mucus, continuous sneezing with runny nose, small pustules on the skin of children by FMPs of Barisal Town in Barisal district [93]; to keep healthy by FMPs of three areas of Pirojpur district [94]; gonorrhea by FMPs of three villages in Panchagarh and Thakurgaon districts [95]; blackening of hair, acne, acidity by FMPs of Vitbilia village of Pabna district [70]; blood purifier, appetite stimulant by FMPs of six villages in Greater Naogaon district [96]; constipation, vomiting by FMPs of seven villages of Ishwardy sub-district in Pabna district [97]; lack of appetite, malaria, hepatitis, sexual disorders, indigestion by FMPs of two villages by the Rupsha River in Bagerhat district [115]; astringent, excessive sexual desire, to increase intelligence, to maintain good eyes, to increase longevity, respiratory problems, coughs, hemorrhoids, leprosy, edema, helminthiasis, breaking down of voice, chronic dysentery, constipation, tumor or swelling, jaundice, loss of appetite, to dissolve stones (like kidney stones) by FMPs of three villages in Sreepur sub-district in Magura district [98]; dysentery, blood dysentery, excessive bleeding during menstruation, jaundice, to ensure proper bowel movement, any type of gastrointestinal disorders by FMPs of Uttar Musrat Madati and Kisasat Madati villages in Lalmonirhat district [60]; to improve functions of liver, stomach and brain, to increase strength and longevity, sex stimulant, pain, rheumatic pain, acne, skin disorders, constipation, hemorrhoids, vomiting by FMPs of Khulna City, Bangladesh [72]; fever by FMPs of Arpara and Munshefpur villages in Jessore district [100]; syphilis, gonorrhea by FMPs of Barobazar village, Jhenidaha district [74]; decreased sperm count, passing of semen with urine by FMPs of Jool chotro and Janga lia villages of Tangail district [75]; fungal infections by FMPs of six villages in Thakurgaon district [76]; vomiting tendency, constipation, skin diseases by the FMP of Kasipur village in Narayanganj district [101]; hemorrhoids by the TMPs of the Baburo, Haduga and Larma clans of the Chakma tribe residing in Rangamati district [53]; coughs, spleen disorders, gastrointestinal disorders by FMPs of Baghbhandar, Sonahat and Kumarpara villages in Kurigram district [102]; gastric problem by FMPs of several villages of Faridpur and Rajbari districts [77]; aphrodisiac, energizer, fever, body ache by the Tonchongya tribal community of Roangchaari sub-district of Bandarban district [49]; urinary tract infection, hysteria by Tripura community of Hazarikhil in Chittagong district [42]; anemia by the Pankho community of Bilaichari Union in Rangamati district [50]; coughs by the Kanda tribe of Sylhet district [51]; coughs by the Rakhaing community of Cox’s Bazar district [52]; hemorrhoids, asthma, coughs, fever by FMPs of Shat-tola Bazaar and Talbari villages in Bagerhat district [79].
*Sansevieria hyacinthoides* (L.) Druce	Ear ache by FMPs and tribal medicinal practitioners (TMPs) of Khakiachora village in Sylhet district [86].
*Jatropha curcas* L.	Swelling of gums by the Goala tribe [65]; white dysentery (presence of mucus in stools) by the Santal tribe residing in Thakurgaon district [111]; dysentery, b lood dysentery, tooth problems by FMPs of Shetabganj village in Dinajpur district [90]; white dysentery by FMPs of three villages in Panchagarh and Thakurgaon districts [95]; dysentery, chronic dysentery by FMPs of Uttar Musrat Madati and Kisasat Madati villages in Lalmonirhat district [60]; irregular menstruation by the TMPs of the Baburo, Haduga and Larma clans of the Chakma tribe residing in Rangamati district [53]; swelling of gums by the Goala tribe of Moulvibazar district [65]; toothache, snake bite, dysentery by the Soren clan of the Santal tribe residing in Nobogram village in Rajshahi district [78]; high blood pressure by Tripura community of Hazarikhil in Chittagong district [42]; lip blister by the Rakhaing community of Cox’s Bazar district [52]; gout, rheumatism, abdominal pain, respiratory problems, edema, obesity, uterine cyst, gall bladder problems, gastrointestinal tract disorders, premature ejaculation, hemorrhoids, ringworm by FMPs of Shat-tola Bazaar and Talbari villages in Bagerhat district [79].
*Phyllanthus emblica* L.	Cardiovascular disorders, hysteria, osteoporosis by the Sardar community [7]; diabetes by the Marakh sect of the Garo tribe [9]; sudden unconsciousness by the Harbang clan of the Tripura tribe [12]; cardiovascular disorders by the Bauri tribe [61]; premature graying of hair by the Goala tribe [65]; diabetes in Dhaka [55]; to increase taste, jaundice, gastric problems, indigestion by the Garo tribe inhabiting Netrakona district [44]; appetite stimulant, indigestion by FMPs of Sylhet Division [83]; to stimulate appetite by the Marma tribal community residing in Naikhongchaari, Bandarban district [45]; appetizer, gonorrhea, toothache, itches by FMPs in five villages of Boalia sub-district, Rajshahi district [116]; burning sensations in urinary tract, leucorrhea, hair loss, reduce graying of hair by FMPs of Shitol Para village, Jhalokati district [66]; alopecia, appetizer by FMPs of villages in Natore and Rajshahi districts [67]; loss of hair, to stop vomiting by FMPs of Daudkandi sub-district in Comilla district [85]; puerperal fever, pain, waist pain, debility by FMPs and tribal medicinal practitioners (TMPs) of Khakiachora village in Sylhet district [86]; erectile dysfunction by FMPs of Bheramara area in Kushtia district [68]; fever, skin problems, loss of appetite, poisonous animal or insect bite, diabetes by the Rakhain tribe of Chittagong Hill Tracts [121]; long-term fever, loss of appetite, sexual stimulant by the TMPs of Tripura tribe residing in Chittagong Hill Tracts [48]; mucus, biliary disorders, loss of appetite, hair loss by the Santal tribe residing in Thakurgaon district [111]; tooth pain by FMPs of Vasu Bihar village, Bogra district [108]; to increase appetite, skin diseases, fever, to increase strength, burning sensations during urination, hair loss, graying of hair by FMPs of Station Purbo Para village in Jamalpur district [89]; hair loss by FMPs of Shetabganj village in Dinajpur district [90]; to increase libido by FMPs of Shekhertek and Badarganj villages in Rangpur district [91]; loss of sensitivity in skin due to allergy or other reasons, chronic mucus, continuous sneezing with runny nose, small pustules on the skin of children by FMPs of Barisal Town in Barisal district [93]; to maintain health by FMPs of three areas of Pirojpur district [94]; gonorrhea by FMPs of three villages in Panchagarh and Thakurgaon districts [95]; to stimulate appetite by FMPs of six villages in Greater Naogaon district [96]; loss of hair, irritation during urination by FMPs of seven villages of Ishwardy sub-district in Pabna district [97]; fistula, lack of appetite, hepatitis, diarrhea, dysentery, cold by FMPs of two villages by the Rupsha River in Bagerhat district [115]; biliary problems, diabetes, alleviation of respiratory, stomach and hepatic problems, fatigue, thirst, burning sensations in the b ody especially in palms of hands or soles of feet, vomiting tendency, insanity by FMPs of three villages in Sreepur sub-district in Magura district [98]; jaundice, blood purifier, anemia, to ensure proper bowel movement, any type of gastrointestinal disorders by FMPs of Uttar Musrat Madati and Kisasat Madati villages in Lalmonirhat district [60]; allergy by FMPs of Fulbaria, Baguri, and Bagh-achra villages in Jessore district [73]; eye infections by FMPs of Barobazar village, Jhenidaha district [74]; syphilis, gonorrhea by FMPs of Barobazar village, Jhenidaha district [74]; decreased sperm count, passing of semen with urine by FMPs of Jool chotro and Janga lia villages of Tangail district [75]; alopecia, indigestion by FMPs of six villages in Thakurgaon district [76]; hemorrhoids by the TMPs of the Baburo, Haduga and Larma clans of the Chakma tribe residing in Rangamati district [53]; blood purifier, anemia, hair loss, coughs, spleen disorders, gastrointestinal disorders by FMPs of Baghbhandar, Sonahat and Kumarpara villages in Kurigram district [102]; anemia by TMPs of a Mro tribal community residing at Gazalia Union of Bandarbans district [112]; stomach troubles, gastric problem by FMPs of several villages of Faridpur and Rajbari districts [77]; graying of hair by the Goala tribe of Moulvibazar district [65]; hair loss by a FMP of Savar in Dhaka district [113]; to increase strength, to clear urine by the Rai Kshatriya tribe of Pabna district [109]; aphrodisiac, energizer, fever, body ache by the Tonchongya tribal community of Roangchaari sub-district of Bandarban district [49]; urinary tract infection, hysteria by Tripura community of Hazarikhil in Chittagong district [42]; coughs, mucus by the Hajong community of Baromari village in Netrakona district [103]; anemia by the Pankho community of Bilaichari Union in Rangamati district [50]; coughs, abdominal gas by the Rakhaing community of Cox’s Bazar district [52]; vaginitis, burning sensations by FMPs of Shat-tola Bazaar and Talbari villages in Bagerhat district [79]; jaundice, to keep head cool, hair loss, graying of hair by folk medicinal herbalists in seven villages of Bhola district [105]; insomnia by the Chakma community of Chittagong Hill Tracts [106].
*Phyllanthus reticulatus* Poir.	Blood dysentery by the Pahan tribe [8]; edema, constipation, cooling of body by FMPs in five villages of Boalia sub-district, Rajshahi district [116]; dysentery by FMPs of Daudkandi sub-district in Comilla district [85]; edema, stomachic, insecticidal, diarrhea, dysentery, bronchitis by FMPs of villages by the Bangali River in Bogra district [59]; used for cooling purposes during excessive heat by FMPs of Vasu Bihar village, Bogra district [108]; stones in gall bladder, kidney or liver, gastrointestinal problems in di8abetic patients by FMPs of Station Purbo Para village in Jamalpur district [89]; chicken pox, swelling of hands or legs by FMPs of seven villages of Ishwardy sub-district in Pabna district [97]; stool color black in children by the Mandai tribe of Bangladesh [117]; dental caries by the Chakma tribe of Hill Tracts districts [118]; chicken pox by FMPs of four villages in Natore and Rajshahi districts [104]; oral lesions in children by folk medicinal herbalists in seven villages of Bhola district [105]; diarrhea in infants by healers among tea garden workers in Sreemangal [119].
*Senna alata* (L.) Roxb.	Eczema, any type of skin disorders by the Harbang clan of the Tripura tribe [12]; skin diseases by the Garo tribe inhabiting Madhupur forest region [62]; skin diseases by FMPs of Noakhali district [64]; eczema by the Goala tribe [65]; ringworm by the Garo tribe inhabiting Netrakona district [44]; ringworm, eczema by the Marma tribal community residing in Naikhongchaari, Bandarban district [45]; ringworm by FMPs of Shitol Para village, Jhalokati district [66]; eczema, wound, helminthiasis, dermatitis, leucorrhea by FMPs of villages in Natore and Rajshahi districts [67]; wet dreams by FMPs of Bheramara area in Kushtia district [68]; ringworm by the TMPs of Tripura tribe residing in Chittagong Hill Tracts [48]; ringworm by FMPs of Balidha village in Jessore district [88]; scabies by FMPs of Station Purbo Para village in Jamalpur district [89]; skin diseases like tinea infections, scabies, herpes by FMPs of Paschim Shawra and Palordi villages of Gaurnadi sub-district in Barisal district [71]; liver diseases by FMPs of six villages in Thakurgaon district [76]; skin diseases by the TMPs of the Baburo, Haduga and Larma clans of the Chakma tribe residing in Rangamati district [53]; eczema by the Goala tribe of Moulvibazar district [65]; stomach pain due to bloating or indigestion by the Tonchongya tribal community residing in Keyaju Para in Bandarban district [124]; ringworm, eczema, itch, scabies, any other skin diseases by the Tonchongya tribal community of Roangchaari sub-district of Bandarban district [49]; eczema by the Chakma tribe of Hill Tracts districts [118]; eczema by Tripura community of Hazarikhil in Chittagong district [42]; sexual disorder by FMPs of four villages in Natore and Rajshahi districts [104]; skin disorders by the Pankho community of Bilaichari Union in Rangamati district [50]; eczema, constipation by the Rakhaing community of Cox’s Bazar district [52]; eczema by the Khatriya and Kashya clans of the Bagdi tribe of Rajbari district [80].
*Hyptis suaveolens* (L.) Poit.	Malaria, headache, insect repellent by the Santal tribe [4]; constipation by the Harbang clan of the Tripura tribe [12]; gonorrhea by the Garo tribe inhabiting Madhupur forest region [62]; constipation by the FMPs of Paikgacha, Khulna district [57]; constipation by FMPs of Sylhet Division [83]; underweight by the Soren clan of the Santal tribe residing in Kannapara and Mondumala villages of Rajshahi district [84]; liver diseases, cancer, constipation by FMPs in five villages of Boalia sub-district, Rajshahi district [116]; stomach problems, to clear objects from eye by FMPs and tribal medicinal practitioners (TMPs) of Khakiachora village in Sylhet district [86]; loss of libido, to keep body cool, boils by FMPs of three villages in Panchagarh and Thakurgaon districts [95]; constipation, dysentery, acidity by the FMP of Kasipur village in Narayanganj district [101]; leucorrhea in women, low sperm density in men by the Bongshi tribe of Tangail district [54]; diabetes, jaundice, burning sensations during urination by the Tonchongya tribal community in Bandarban district [124]; cooling agent, kidney disease, urinary tract infections, dysuria, laxative by the Tonchongya tribal community of Roangchaari sub-district of Bandarban district [49]; fever, urinary complications by the Chakma tribe of Hill Tracts districts [118]; flatulence, acidity, gastric troubles by the Hajong community of Baromari village in Netrakona district [103]; stomach ache in children by the Pankho community of Bilaichari Union in Rangamati district [50]; fever by the Chakma community of Chittagong Hill Tracts [106].
*Leucas aspera* (Willd.) Link	Severe headache due to fever, dog bite by the Kole tribe [10]; skin disorders, failure to conceive by the folk medicinal practitioners (FMPs) of Jessore district [63]; dysentery, external bleeding, eye inflammation by FMPs of Noakhali district [64]; colic by the FMPs of Rampal in Bagerhat district [57]; treatment of gastrointestinal and hepatic disorders by FMPs of Bagerhat Sadar in Bagerhat district [58]; diarrhea, blood purifier, indigestion, loss of appetite by FMPs in Tangail district [82]; scabies, skin infections by FMPs of Sylhet Division [83]; chronic coughs by the Marma tribal community residing in Naikhongchaari, Bandarban district [45]; peptic ulcer, stomach ache by FMPs of Daudkandi sub-district in Comilla district [85]; tooth infection, mucus by FMPs of Dhamrai sub-district of Dhaka district [87]; skin problems, scabies, eczema, rheumatic pain, joint pain, coughs, mucus, throat pain, pain or inflammation of or within the body, lesions within the nose, pain or bleeding from the nose by the Rakhain tribe of Chittagong Hill Tracts [121]; headache by the ojhas (tribal medicinal practitioners) of the Santal tribe of Rajshahi district [110]; coughs, blood with cough, colds and associated problems by the TMPs of Tripura tribe residing in Chittagong Hill Tracts [48]; bone fracture, leprosy by FMPs of Rahmatpur village by the Ghaghot River, Rangpur district [59]; gout by FMPs of villages by the Padma River in Rajshahi district [59]; gout by FMPs of Vasu Bihar village, Bogra district [108]; pain in bones, body pain, asthma by FMPs of Station Purbo Para village in Jamalpur district [89]; sudden feeling of warmth in head, headache by FMPs of Shekhertek and Badarganj villages in Rangpur district [91]; excessive salivating by FMPs of Daulatdia Ghat in Kushtia district [92]; pain with accompanied swelling, goiter in women, respiratory difficulties by FMPs of Barisal Town in Barisal district [93]; diarrhea, blood purifier, loss of appetite, indigestion by FMPs of Babla and Terbaria villages in Tangail district [82]; snake bite by FMPs of Shonapur, Chorkulte, and Majhbari villages of Rajbari district [99]; headache by FMPs of Jool chotro and Janga lia villages of Tangail district [75]; lesions/infections within nostrils by the TMPs of the Baburo, Haduga and Larma clans of the Chakma tribe residing in Rangamati district [53]; severe pain by FMPs of Baghbhandar, Sonahat and Kumarpara villages in Kurigram district [102]; pain in one side of the head, helminthiasis by the Bongshi tribe of Tangail district [54]; severe pain, rheumatic pain by FMPs of several villages of Faridpur and Rajbari districts [77]; constipation by the Naik clan of the Rajbongshi tribe of Moulvibazar district [46]; common cold by a FMP of Savar in Dhaka district [113]; excessive menstrual bleeding by the Tonchongya tribal community of Roangchaari sub-district of Bandarban district [49]; tonsillitis, mumps by FMPs of four villages in Natore and Rajshahi districts [104]; lesions on the tongue, pain due to hemorrhoids by the Murmu tribal community of Rajshahi district [123]; coughs in infants, throat pain by healers among tea garden workers in Sreemangal [119].
*Ocimum gratissimum* L.	Eczema, cough, mucus by the Harbang clan of the Tripura tribe [12]; heart diseases, eye diseases, cooling of body by FMPs in five villages of Boalia sub-district, Rajshahi district [116]; chest pain, rhinitis, coughs, itching, helminthiasis, ear ache, poisonous snake or reptile bite by FMPs of Shitol Para village, Jhalokati district [66]; coughs, appetizer, dysentery, fever by FMPs of villages in Natore and Rajshahi districts [67]; coughs, fever by FMPs of Daudkandi sub-district in Comilla district [85]; colds, coughs by FMPs of Bheramara area in Kushtia district [68]; fever with convulsions, burning sensations in the body, indigestion, pneumonia, coughs, mucus, tingling sensations in the body by the TMPs of Tripura tribe residing in Chittagong Hill Tracts [48]; rabies, pneumonia by FMPs of Rahmatpur village by the Ghaghot River, Rangpur district [59]; coughs, cold by FMPs of villages by the Padma River in Rajshahi district [59]; cold by FMPs of five villages in Narsinghdi district [69]; rheumatic pain by FMPs of Shetabganj village in Dinajpur district [90]; coughs, mucus by FMPs of Shekhertek and Badarganj villages in Rangpur district [91]; asthma, bronchitis by FMPs of Vitbilia village of Pabna district [70]; chest pain, coughs, respiratory tract infections by FMPs of Paschim Shawra and Palordi villages of Gaurnadi sub-district in Barisal district [71]; fever, coughs, carminative by FMPs of Uttar Musrat Madati and Kisasat Madati villages in Lalmonirhat district [60]; coughs by FMPs of Arpara and Munshefpur villages in Jessore district [100]; coughs by FMPs of six villages in Thakurgaon district [76]; fever wiyh pain by FMPs of Baghbhandar, Sonahat and Kumarpara villages in Kurigram district [102].
*Allium sativum* L.	Infertility with seizures by the Hodi tribe [6]; diabetes in Dhaka [55]; indigestion, sedative, impotency by FMPs of villages in Natore and Rajshahi districts [67]; rabies, pneumonia by FMPs of Rahmatpur village by the Ghaghot River, Rangpur district [59]; cold, hair loss, diabetes by FMPs of Station Purbo Para village in Jamalpur district [89]; diabetes by FMPs of Daulatdia Ghat in Kushtia district [92]; rheumatism by FMPs of four adjoining villages in Narail and Jessore districts [122]; diabetes, jaundice, rheumatism, rheumatic pain, paralysis by FMPs of Barobazar village, Jhenidaha district [74]; joint pain by the FMP of Kasipur village in Narayanganj district [101]; rheumatic pain, passing of semen with urine by FMPs of several villages of Faridpur and Rajbari districts [77]; rheumatic pain by the Naik clan of the Rajbongshi tribe of Moulvibazar district [46]; to reduce fat in the body by a FMP of Savar in Dhaka district [113]; paralysis, chest cold by the Rai Kshatriya tribe of Pabna district [109]; coughs in children by Tripura community of Hazarikhil in Chittagong district [42]; high blood pressure, leprosy by the Rakhaing community of Cox’s Bazar district [52]; stomach disorders by folk medicinal herbalists in seven villages of Bhola district [105]; leprosy, whooping cough, blood pressure by the Chakma community of Chittagong Hill Tracts [106].
*Lawsonia inermis* L.	Bloating by the Harbang clan of the Tripura tribe [12]; eczema, leprosy, jaundice by the Garo tribe inhabiting Netrakona district [44]; cancer, fever, burning sensations during urination, to keep head cool by FMPs of Sylhet Division [83]; jaundice, swelling and burning sensations due to burns caused from fire, dandruff by FMPs of Shitol Para village, Jhalokati district [66]; diabetes, heart diseases, blood dysentery by FMPs of villages in Natore and Rajshahi districts [67]; hair loss, leprosy by FMPs of Daudkandi sub-district in Comilla district [85]; diabetes by FMPs and tribal medicinal practitioners (TMPs) of Khakiachora village in Sylhet district [86]; leprosy by FMPs of Rahmatpur village by the Ghaghot River, Rangpur district [59]; leg infections, toe nail infections by FMPs of villages by the Padma River in Rajshahi district [59]; to keep head cool, dandruff in humans, broken shoulder in cattle by FMPs of Station Purbo Para village in Jamalpur district [89]; weakness by FMPs of three areas of Pirojpur district [94]; itch, ear ache by FMPs of three villages in Panchagarh and Thakurgaon districts [95]; gonorrhea, jaundice by FMPs of six villages in Greater Naogaon district [96]; anti-dandruff, antiseptic, burns by FMPs of seven villages of Ishwardy sub-district in Pabna district [97]; to keep head cool by FMPs of Paschim Shawra and Palordi villages of Gaurnadi sub-district in Barisal district [71]; eczema, skin diseases by FMPs of Uttar Musrat Madati and Kisasat Madati villages in Lalmonirhat district [60]; diabetes by FMPs of Fulbaria, Baguri, and Bagh-achra villages in Jessore district [73]; excessive bleeding during menstruation by FMPs of Arpara and Munshefpur villages in Jessore district [100]; eye infections by FMPs of Barobazar village, Jhenidaha district [74]; dandruff, skin diseases, sexually transmitted diseases by the FMP of Kasipur village in Narayanganj district [101]; skin infections, blood coming out of mouth, pus in ears by FMPs of Baghbhandar, Sonahat and Kumarpara villages in Kurigram district [102]; severe pain by the Bongshi tribe of Tangail district [54]; emollient, hair conditioner by FMPs of four villages in Natore and Rajshahi districts [104].
*Melastoma malabathricum* L.	Urinary problems by the Garo tribe inhabiting Netrakona district [44]; jaundice by the Marma tribal community residing in Naikhongchaari, Bandarban district [45]; low sperm count, sperm with urine, jaundice, pain by the Rakhain tribe of Chittagong Hill Tracts [121]; any sort of body pain, diarrhea, dysentery, scabies, abscess, leucorrhea, urinary problems by the TMPs of Tripura tribe residing in Chittagong Hill Tracts [48]; urinary tract infection by the Tonchongya tribal community of Roangchaari sub-district of Bandarban district [49].
*Azadirachta indica* A. Juss.	To induce fertility, cancer, acne, itch, carminative by the Santal tribe [4]; tooth infections by the Sardar community [7]; blood dysentery, piles by the Pahan tribe [8]; cuts and wounds, allergy, premature graying of hair by the Khasia tribe [11]; skin diseases, helminthiasis by the Harbang clan of the Tripura tribe [12]; by the Bauri tribe [61]; skin disorders by the folk medicinal practitioners (FMPs) of Jessore district [63]; stomach and heart disorders by the Goala tribe [65]; diabetes in Dhaka [55]; diarrhea, blood purifier, indigestion, loss of appetite by FMPs in Tangail district [82]; fever, chicken pox, measles, skin diseases by the Garo tribe inhabiting Netrakona district [44]; cancer, skin diseases, helminthiasis, wounds, diabetes, rheumatoid arthritis by FMPs in five villages of Boalia sub-district, Rajshahi district [116]; itches, scabies, allergy, pus formation, skin disorders by FMPs of Shitol Para village, Jhalokati district [66]; dental diseases, scabies by FMPs of Daudkandi sub-district in Comilla district [85]; diabetes by FMPs and tribal medicinal practitioners (TMPs) of Khakiachora village in Sylhet district [86]; fever, fever arising out from gall bladder disorders by FMPs of Bheramara area in Kushtia district [68]; abscess by the Santal tribe residing in Thakurgaon district [111]; syphilis, skin diseases, scabies, leprosy by FMPs of Rahmatpur village by the Ghaghot River, Rangpur district [59]; helminthiasis, itches by FMPs of villages by the Padma River in Rajshahi district [59]; coughs, respiratory illnesses by FMPs of five villages in Narsinghdi district [69]; insecticide by FMPs of Vasu Bihar village, Bogra district [108]; considered useful in any type of disease by FMPs of Balidha village in Jessore district [88]; skin diseases, body ache, bone pain, measles, pox, itches, scabies, indigestion, cataract, decreased eye sight, abscess by FMPs of Station Purbo Para village in Jamalpur district [89]; helminthiasis, hepatic pain by FMPs of Shetabganj village in Dinajpur district [90]; scabies, eczema, itches by FMPs of Shekhertek and Badarganj villages in Rangpur district [91]; irregular menstruation, diabetes by FMPs of Daulatdia Ghat in Kushtia district [92]; diabetes by FMPs of Barisal Town in Barisal district [93]; all types of pain, fever by FMPs of three villages in Panchagarh and Thakurgaon districts [95]; antiseptic, skin diseases, helminthiasis, pimples, acidity, blood purifier by FMPs of seven villages of Ishwardy sub-district in Pabna district [97]; diarrhea, blood purifier, loss of appetite, indigestion by FMPs of Babla and Terbaria villages in Tangail district [82]; good for eyes, to increase lung capacity, fatigue, thirsts, coughs, fever, loss of appetite, helminthiasis, acne, biliary disorders, leprosy, wasting away of body, diabetes by FMPs of three villages in Sreepur sub-district in Magura district [98]; skin diseases like scabies and eczema, gum diseases by FMPs of Paschim Shawra and Palordi villages of Gaurnadi sub-district in Barisal district [71]; fever, body pain, swelling of knees, injury, rheumatic pain, itches, scabies, infections, considered beneficial in nearly all types of diseases, skin diseases, pyorrhea by FMPs of Uttar Musrat Madati and Kisasat Madati villages in Lalmonirhat district [60]; scabies, itches by FMPs of Khulna City, Bangladesh [72]; allergy by FMPs of Fulbaria, Baguri, and Bagh-achra villages in Jessore district [73]; skin diseases, leucorrhea by FMPs of Arpara and Munshefpur villages in Jessore district [100]; toothache by FMPs of six villages in Thakurgaon district [76]; skin diseases, tooth infections, helminthiasis by the FMP of Kasipur village in Narayanganj district [101]; fever, pain, itches, rheumatic pain, skin infections, bleeding from gums, swelling of gums, tingling sensation in gums, foul odor in mouth by FMPs of Baghbhandar, Sonahat and Kumarpara villages in Kurigram district [102]; skin diseases, allergy by the Bongshi tribe of Tangail district [54]; severe pain, rheumatic pain by FMPs of several villages of Faridpur and Rajbari districts [77]; stomach and heart disorders by the Goala tribe of Moulvibazar district [65]; to strengthen base of tooth, acne by a FMP of Savar in Dhaka district [113]; paralysis, skin infections by the Rai Kshatriya tribe of Pabna district [109]; blood purifier by the Soren clan of the Santal tribe residing in Nobogram village in Rajshahi district [78]; scabies, itches by the Sigibe clan of the Khumi tribe residing in Thanchi sub-district of Bandarban district [47]; toothache, itches by the Hajong community of Baromari village in Netrakona district [103]; itches, pain by FMPs of four villages in Natore and Rajshahi districts [104]; diabetes by the Pankho community of Bilaichari Union in Rangamati district [50]; helminthiasis by the Kanda tribe of Sylhet district [51]; chicken pox, high blood pressure, gastritis, general weakness, jaundice, malaria by the Rakhaing community of Cox’s Bazar district [52]; fever, pain, to prevent tooth infections by the Murmu tribal community of Rajshahi district [123]; pimple, tiredness, coughs, vomiting, gall bladder problems, helminthiasis, pain, dyspepsia, leprosy, acne, gleet, gonorrhea, diabetes by FMPs of Shat-tola Bazaar and Talbari villages in Bagerhat district [79]; skin disorders, tooth infections, foul odor in mouth by the Tripura tribe residing in Comilla district [81].
*Ficus hispida* L.	Insect repellent, diabetes, analgesic by the Santal tribe[4]; diabetes, weakness by the folk medicinal practitioners (FMPs) of Jessore district [63]; snake bite by the Goala tribe [65]; gall bladder diseases by FMPs of Bheramara area in Kushtia district [68]; dermatitis, carminative, stomachic (for cattle) by FMPs of villages by the Bangali River in Bogra district [59]; dysentery by FMPs of Station Purbo Para village in Jamalpur district [89]; internal bleeding by FMPs of Daulatdia Ghat in Kushtia district [92]; dysentery by FMPs of Barisal Town in Barisal district [93]; diabetes by FMPs of three villages in Panchagarh and Thakurgaon districts [95]; whooping cough by FMPs of six villages in Greater Naogaon district [96]; persistent itching, skin irritation, swelling, inflammation by FMPs of Paschim Shawra and Palordi villages of Gaurnadi sub-district in Barisal district [71]; physical weakness, malnutrition, leucorrhea, to increase digestion, constipation, flatulence by FMPs of Khulna City, Bangladesh [72]; diabetes, leucorrhea by FMPs of Fulbaria, Baguri, and Bagh-achra villages in Jessore district [73]; to keep healthy, diabetes by FMPs of six villages in Thakurgaon district [76]; to aid digestion, constipation by the FMP of Kasipur village in Narayanganj district [101]; diabetes by the TMPs of the Baburo, Haduga and Larma clans of the Chakma tribe residing in Rangamati district [53]; diabetes by FMPs of Baghbhandar, Sonahat and Kumarpara villages in Kurigram district [102]; snake bite by the Goala tribe of Moulvibazar district [65]; hookworm by the Tonchongya tribal community residing in Keyaju Para in Bandarban district [124]; anxiolytic by FMPs of four villages in Natore and Rajshahi districts [104]; diabetes by folk medicinal herbalists in seven villages of Bhola district [105].
*Moringa oleifera* Lam.	Constipation, epilepsy, abortifacient, skin eruptions, leucoderma by the Santal tribe [4]; rheumatism, chicken pox, snake repellent by the Pahan tribe [8]; nasal catarrh, decreased eye sight, bone fractures, sores by the Garo tribe inhabiting Madhupur forest region [62]; skin lesions, cancer, chicken pox by the folk medicinal practitioners (FMPs) of Jessore district [63]; diabetes by the Garo tribe inhabiting Netrakona district [44]; sex stimulant, headache, coughs, mucus by FMPs of Sylhet Division [83]; paralysis by FMPs of a group of Christians residing in Mirzapore village of Dinajpur district [107]; appetite stimulant, carminative, heart disorders, rheumatic fever, paralysis, liver pain, to increase bile secretion, sex stimulant by FMPs of Shitol Para village, Jhalokati district [66]; hypertension, rheumatoid arthritis, leprosy, conjunctivitis, pain by FMPs of villages in Natore and Rajshahi districts [67]; sterility by FMPs of Daudkandi sub-district in Comilla district [85]; puerperal fever, pain, jaundice by FMPs and tribal medicinal practitioners (TMPs) of Khakiachora village in Sylhet district [86]; contraceptive, gout by FMPs of villages by the Padma River in Rajshahi district [59]; helminthiasis by FMPs of Vasu Bihar village, Bogra district [108]; rheumatism, ear disease, headache by FMPs of Balidha village in Jessore district [88]; diabetes, acidity, hypertension by FMPs of Station Purbo Para village in Jamalpur district [89]; hemorrhoids by FMPs of three areas of Pirojpur district [94]; high blood pressure by FMPs of three villages in Panchagarh and Thakurgaon districts [95]; paralysis, body pain by FMPs of six villages in Greater Naogaon district [96]; to stimulate appetite, roughness of skin, to increase sperm, helminthiasis, obesity, coughs, restless feeling, bloating, swelling due to injury, formation of blood clots on skin, goiter, acne, good for eyes, pain, headache by FMPs of three villages in Sreepur sub-district in Magura district [98]; diabetes, frequent urination by FMPs of Shonapur, Chorkulte, and Majhbari villages of Rajbari district [99]; small pox, chicken pox, anemia by FMPs of Barobazar village, Jhenidaha district [74]; diabetes, acidity, hypertension by FMPs of six villages in Thakurgaon district [76]; hypertension, swelling of gums, malnutrition by FMPs of Baghbhandar, Sonahat and Kumarpara villages in Kurigram district [102]; hepatitis, jaundice by FMPs of several villages of Faridpur and Rajbari districts [77]; constipation, liver problems, joint pain by a FMP of Savar in Dhaka district [113]; diabetes by the Rai Kshatriya tribe of Pabna district [109]; indigestion by FMPs of four villages in Natore and Rajshahi districts [104]; conjunctivitis by the Kanda tribe of Sylhet district [51]; burning, general weakness, headache, insomnia, high blood pressure, leucorrhea by the Rakhaing community of Cox’s Bazar district [52]; to stimulate appetite, roughness of skin, pain, to increase sperm, acne, helminthiasis, obesity, coughs, flatulence, restless feeling, swelling due to injury, formation of blood clots on skin, goiter, headache by FMPs of Shat-tola Bazaar and Talbari villages in Bagerhat district [79].
*Psidium guajava* L.	Diabetes, toothache, carminative by the Santal tribe [4]; diarrhea, debility by FMPs in Tangail district [82]; toothache, acne, diabetes by the Garo tribe inhabiting Netrakona district [44]; toothache, diarrhea by FMPs of Sylhet Division [83]; dental pain, gingivitis, scabies by FMPs of Shitol Para village, Jhalokati district [66]; hemorrhoids by FMPs of Daudkandi sub-district in Comilla district [85]; diabetes by FMPs and tribal medicinal practitioners (TMPs) of Khakiachora village in Sylhet district [86]; dysentery, coughs, mucus, cold, wounds, respiratory problems, maintain texture of skin, maintain normal heart condition by the Rakhain tribe of Chittagong Hill Tracts [121]; dysentery by FMPs of villages by the Padma River in Rajshahi district [59]; gastric problems, cuts and wounds by FMPs of Balidha village in Jessore district [88]; menstrual problems, diarrhea, tooth infections by FMPs of Station Purbo Para village in Jamalpur district [89]; dysentery, anorexia by FMPs of three areas of Pirojpur district [94]; diarrhea by FMPs of six villages in Greater Naogaon district [96]; diarrhea, debility by FMPs of Babla and Terbaria villages in Tangail district [82]; uterine prolapse by FMPs of four adjoining villages in Narail and Jessore districts [122]; loss of libido in men, puerperal fever by FMPs of Uttar Musrat Madati and Kisasat Madati villages in Lalmonirhat district [60]; dysentery, to strengthen teeth by FMPs of Shonapur, Chorkulte, and Majhbari villages of Rajbari district [99]; dysentery by FMPs of Jool chotro and Janga lia villages of Tangail district [75]; menstrual problems by FMPs of six villages in Thakurgaon district [76]; dysentery by the FMP of Kasipur village in Narayanganj district [101]; dysentery, puerperal fever by FMPs of Baghbhandar, Sonahat and Kumarpara villages in Kurigram district [102]; dysentery by Tripura community of Hazarikhil in Chittagong district [42]; for removing stains from teeth by FMPs of four villages in Natore and Rajshahi districts [104]; to increase strength, sperm count and appetite, hemorrhoids by FMPs of Shat-tola Bazaar and Talbari villages in Bagerhat district [79].
*Plumbago indica* L.	To stop bleeding from cuts and wounds by the Pahan tribe [8]; abortifacient, leprosy, paralysis, piles, constriction of nerves leading to distortions in hands or feet, stoppage of urination, paralysis by the Soren clan of the Santal tribe residing in Kannapara and Mondumala villages of Rajshahi district [84]; vitiligo by FMPs of Rahmatpur village by the Ghaghot River, Rangpur district [59]; memory enhancer, hemorrhoids by FMPs of villages by the Padma River in Rajshahi district [59]; blood purifier by FMPs of Paschim Shawra and Palordi villages of Gaurnadi sub-district in Barisal district [71]; stomach pain by the Kanda tribe of Sylhet district [51]; hydrocele by the Khatriya and Kashya clans of the Bagdi tribe of Rajbari district [80]; dysentery by the Chakma community of Chittagong Hill Tracts [106].
*Persicaria glabra* (Willd.) M. Gómez	Insect bites, abscess by FMPs of several areas of Faridpur and Rajbari districts [77].
*Eichhornia crassipes* (Mart.) Solms	Snake bite, hives by the Santal tribe [4]; hepatic disorders, swelling of one side of abdomen by FMPs of a group of Christians residing in Mirzapore village of Dinajpur district [107]; insecticidal, astringent by FMPs of villages by the Bangali River in Bogra district [59]; fever by FMPs of six villages in Thakurgaon district [76]; asthma by Tripura community of Hazarikhil in Chittagong district [42].
*Paederia foetida* L.	Insanity, mental disorders by the Rai tribe [10]; stomach ailments by the Garo tribe inhabiting Madhupur forest region [62]; loss of appetite, indigestion, diarrhea, dysentery, weakness, toothache, cancer by FMPs of Noakhali district [64]; bloating, indigestion by FMPs of Sylhet Division [83]; dyspepsia, constipation, cholera, dysentery of domestic animals by FMPs of Shitol Para village, Jhalokati district [66]; tonic, rheumatoid arthritis, colic by FMPs of villages in Natore and Rajshahi districts [67]; indigestion, stomach ache by FMPs of Balidha village in Jessore district [88]; bloating by FMPs of Station Purbo Para village in Jamalpur district [89]; coughs, mucus, loss of appetite, rheumatism, pain, dysentery by FMPs of Daulatdia Ghat in Kushtia district [92]; any type of pain by FMPs of three villages in Panchagarh and Thakurgaon districts [95]; body pain by FMPs of six villages in Greater Naogaon district [96]; internal lesions, stomach problems, to recuperate from illness by FMPs of seven villages of Ishwardy sub-district in Pabna district [97]; fractures, to increase strength, to increase sperm, rheumatism, pain, hemorrhoids, skin allergy, constipation by FMPs of three villages in Sreepur sub-district in Magura district [98]; chronic dysentery, dysentery by FMPs of Uttar Musrat Madati and Kisasat Madati villages in Lalmonirhat district [60]; indigestion by FMPs of six villages in Thakurgaon district [76]; rheumatic pain, burning sensations during urination by the TMPs of the Baburo, Haduga and Larma clans of the Chakma tribe residing in Rangamati district [53]; dysentery by FMPs of Baghbhandar, Sonahat and Kumarpara villages in Kurigram district [102]; toothache by the Bongshi tribe of Tangail district [54]; appetizer by FMPs of four villages in Natore and Rajshahi districts [104].
*Aegle marmelos* (L.) Corr.	Anti-inflammatory, constipation, blood dysentery, diabetes by the Santal tribe [4]; to keep head cool, sprain, fracture by the Rai tribe [10]; indigestion, loss of appetite, constipation, weakness, dysentery, snake bite, skin infections by FMPs of Noakhali district [64]; constipation, dysentery, indigestion, pain by the Garo tribe inhabiting Netrakona district [44]; urinary bladder stone by FMPs of Sylhet Division [83]; sedative by the Marma tribal community residing in Naikhongchaari, Bandarban district [45]; pain under the umbilicus and stomach ache due to worms, constipation, decreased sperm count, aphrodisiac by FMPs of Shitol Para village, Jhalokati district [66]; indigestion, cooling of body, appetizer, loss of libido, weakness, paralysis by FMPs of villages in Natore and Rajshahi districts [67]; dysentery, peptic ulcer by FMPs of Daudkandi sub-district in Comilla district [85]; puerperal fever, pain, waist pain, debility, jaundice, blood with stool, stomach ache by FMPs and tribal medicinal practitioners (TMPs) of Khakiachora village in Sylhet district [86]; indigestion, hemorrhoids, constipation, respiratory problems, inflammation, poisonous insect or snake bite, heart palpitations, fever, cleaning of bowels by the Rakhain tribe of Chittagong Hill Tracts [121]; to keep body cool, diarrhea, dysentery, constipation, stringent, repeat fevers, contagious fevers, frequent urination (diabetes) by the TMPs of Tripura tribe residing in Chittagong Hill Tracts [48]; flatulence by FMPs of Rahmatpur village by the Ghaghot River, Rangpur district [59]; liver disorder, sun stroke, jaundice, constipation, sexual disorder, hemorrhoids, apepsia (in cattle) by FMPs of villages by the Bangali River in Bogra district [59]; dysentery by FMPs of Vasu Bihar village, Bogra district [108]; excessive blood during menstruation, diseases of the scrotum by FMPs of Balidha village in Jessore district [88]; chronic dysentery, diabetes by FMPs of Station Purbo Para village in Jamalpur district [89]; to remove foul odor of sweat, vomiting in children by FMPs of Shetabganj village in Dinajpur district [90]; to remove odor from sweat, incoherency or insanity, acidity, ear diseases, eye diseases by FMPs of Daulatdia Ghat in Kushtia district [92]; chronic dysentery, constipation, indigestion by FMPs of three areas of Pirojpur district [94]; digestive aid by FMPs of six villages in Greater Naogaon district [96]; acidity, skin allergy, excessive sexual desire, carminative, coughs, astringent by FMPs of three villages in Sreepur sub-district in Magura district [98]; uterine prolapse by FMPs of four adjoining villages in Narail and Jessore districts [122]; long-term fever with abnormally high body temperature by FMPs of Paschim Shawra and Palordi villages of Gaurnadi sub-district in Barisal district [71]; blood dysentery, spermatorrhea, snake bite, to increase functions of brain, stomach, heart and liver, diarrhea, dysentery, low density of semen, insomnia, weakness of heart, mucus, fever with mucus, excessive thirst, vomiting, constipation by FMPs of Khulna City, Bangladesh [72]; dysentery by FMPs of Barobazar village, Jhenidaha district [74]; dysentery, to remove foul odor from sweat, insanity, acidity, ear diseases, eye diseases by FMPs of Jool chotro and Janga lia villages of Tangail district [75]; dysentery, constipation, excessive bleeding during menstruation by FMPs of six villages in Thakurgaon district [76]; blood dysentery, to increase memory, constipation, to prevent stomach upsets by the FMP of Kasipur village in Narayanganj district [101]; flatulence, constipation, stomach pain by the TMPs of the Baburo, Haduga and Larma clans of the Chakma tribe residing in Rangamati district [53]; dysentery, to remove odor of sweat by FMPs of Baghbhandar, Sonahat and Kumarpara villages in Kurigram district [102]; severe pain, gastric problem by FMPs of several villages of Faridpur and Rajbari districts [77]; constipation by the Naik clan of the Rajbongshi tribe of Moulvibazar district [46]; acne by a FMP of Savar in Dhaka district [113]; sexual disorder in males by the Rai Kshatriya tribe of Pabna district [109]; flatulence by the Soren clan of the Santal tribe residing in Nobogram village in Rajshahi district [78]; stomach pain, dysentery with blood by Tripura community of Hazarikhil in Chittagong district [42]; chronic dysentery by FMPs of four villages in Natore and Rajshahi districts [104]; vomiting by the Rakhaing community of Cox’s Bazar district [52]; jaundice, indigestion by the Murmu tribal community of Rajshahi district [123]; to increase digestive capability, coughs, flatulence, to keep body cool, to clear stool by FMPs of Shat-tola Bazaar and Talbari villages in Bagerhat district [79]; dysentery, diarrhea by the Tripura tribe residing in Comilla district [81]; dysentery, to keep stomach cool, sudden bouts of vomiting by folk medicinal herbalists in seven villages of Bhola district [105]; dysentery, diarrhea by the Chakma community of Chittagong Hill Tracts [106].
*Santalum album* L.	Tuberculosis, debility, burning sensations during urination by FMPs of Noakhali district [64]; loss of sexual desire, to induce a satisfactory feeling in body, slight edema, mucus, biliary disorders, blood purifier, burning sensations in the body by FMPs of three villages in Sreepur sub-district in Magura district [98]; leucorrhea, sexual weakness by FMPs of Paschim Shawra and Palordi villages of Gaurnadi sub-district in Barisal district [71]; headache, chronic coughs, discoloration of facial skin, gonorrhea, abnormal heart palpitations, hypertension, b lood purifier by FMPs of Khulna City, Bangladesh [72]; dysentery by FMPs of six villages in Thakurgaon district [76]; to remove scar marks or marks due to burns, skin diseases by the TMPs of the Baburo, Haduga and Larma clans of the Chakma tribe residing in Rangamati district [53]; mucus by TMPs of a Mro tribal community residing at Gazalia Union of Bandarbans district [112]; cough by Tripura community of Hazarikhil in Chittagong district [42]; eczema, coughs by the Rakhaing community of Cox’s Bazar district [52].
*Scoparia dulcis* L.	Coughs in children, diarrhea by the Harbang clan of the Tripura tribe [12]; dysentery by the Garo tribe inhabiting Madhupur forest region [62]; diabetes, cuts and wounds, gastric ulcer, weakness, fever, coughs, bronchitis, diarrhea, dysentery, edema, diabetes, toothache by FMPs of Noakhali district [64]; diarrhea, dysentery and colic by FMPs of Rampal in Bagerhat district, and for constipation by FMPs of Paikgacha in Khulna district [57]; sexual disorders by FMPs of Bagerhat Sadar in Bagerhat district [58]; urinary problems by FMPs in Tangail district [82]; sexual diseases, nerve disorders by FMPs of Sylhet Division [83]; respiratory problems, to stimulate appetite by the Marma tribal community residing in Naikhongchaari, Bandarban district [45]; ulcer by FMPs and tribal medicinal practitioners (TMPs) of Khakiachora village in Sylhet district [86]; diabetes by FMPs of Dhamrai sub-district of Dhaka district [87]; blood dysentery by the ojhas (tribal medicinal practitioners) of the Santal tribe of Rajshahi district [110]; diabetes by the Santal tribe residing in Thakurgaon district [111]; infertility, leucorrhea, malaria, dog bite, debility, hemorrhoids by FMPs of villages by the Bangali River in Bogra district [59]; debility, premature ejaculation by FMPs of villages by the Padma River in Rajshahi district [59]; gastric ulcer by FMPs of five villages in Narsinghdi district [69]; diabetes by FMPs of Station Purbo Para village in Jamalpur district [89]; urinary problems by FMPs of Shetabganj village in Dinajpur district [90]; continuous hiccups by FMPs of Shekhertek and Badarganj villages in Rangpur district [91]; dysentery in children by FMPs of Barisal Town in Barisal district [93]; body ache, gastric ulcer by FMPs of three areas of Pirojpur district[94]; burning sensations during urination by FMPs of six villages in Greater Naogaon district [96]; gastric ulcer, anemia by FMPs of seven villages of Ishwardy sub-district in Pabna district [97]; urinary problems by FMPs of Babla and Terbaria villages in Tangail district [82]; aphrodisiac, sexual disorders, dysentery, diabetes by FMPs of Paschim Shawra and Palordi villages of Gaurnadi sub-district in Barisal district [71]; dysentery, white dysentery (passing of mucus with stool), any injury causing pain by FMPs of Fulbaria, Baguri, and Bagh-achra villages in Jessore district [73]; appetite stimulant, diabetes, dysentery by FMPs of Shonapur, Chorkulte, and Majhbari villages of Rajbari district [99]; leucorrhea by FMPs of Barobazar village, Jhenidaha district [74]; gastric problems, ulcer by FMPs of six villages in Thakurgaon district [76]; pain in chin or throat, tonsillitis, throat cancer, facial redness, skin diseases by the TMPs of the Baburo, Haduga and Larma clans of the Chakma tribe residing in Rangamati district [53]; ‘meho’ by FMPs of Baghbhandar, Sonahat and Kumarpara villages in Kurigram district [102]; diarrhea in children by the Bongshi tribe of Tangail district [54]; gastric problems, dysentery, diabetes by a FMP of Savar in Dhaka district [113]; jaundice by the Rai Kshatriya tribe of Pabna district [109]; snake and insect bite, antidote to poison by the Tonchongya tribal community of Roangchaari sub-district of Bandarban district [49]; spermatorrhea by the Pankho community of Bilaichari Union in Rangamati district [50]; stomach ache in infants by healers among tea garden workers in Sreemangal [119].
*Smilax macrophylla* Roxb.	Rheumatism, body pain by FMPs of two areas of Dinajpur district [125].
*Physalis micrantha* Link	Ear ache by the Khatriya and Kashya clans of the Bagdi tribe of Rajbari district [80].
*Pouzolzia zeylanica* (L.) Benn.	
*Clerodendrum viscosum* Vent.	Skin eruption, fever, dysentery by the Santal tribe [4]; diabetes by the Marakh sect of the Garo tribe [9]; feeling of weakness during times of menstruation by the Harbang clan of the Tripura tribe [12]; colic pain by the Garo tribe inhabiting Madhupur forest region [62]; intestinal worms by the folk medicinal practitioners (FMPs) of Jessore district [63]; coughs, asthma, skin diseases, snake bite, gonorrhea, low semen density, leucorrhea by FMPs of Noakhali district [64]; pain in body, blood purifier by FMPs in Tangail district [82]; lice infections by the Garo tribe inhabiting Netrakona district [44]; coughs in children by FMPs of Sylhet Division [83]; helminthiasis, gastric ulcer by FMPs of a group of Christians residing in Mirzapore village of Dinajpur district [107]; nausea, vomiting, puerperal fever by FMPs of Shitol Para village, Jhalokati district [66]; tonic, gastritis, dermatitis, dysentery by FMPs of villages in Natore and Rajshahi districts [67]; diabetes by FMPs and tribal medicinal practitioners (TMPs) of Khakiachora village in Sylhet district [86]; fever in children, toothache, pain in gums by FMPs of Dhamrai sub-district of Dhaka district [87]; helminthiasis, toothache, lesions within the ear, fever with convulsions, malaria by the Rakhain tribe of Chittagong Hill Tracts [121]; stomach pain, acidity, redness of eye, malarial fever, fever, coughs, helminthiasis, respiratory problems by the TMPs of Tripura tribe residing in Chittagong Hill Tracts [48]; helminthiasis, infections from scorpion bites by the Santal tribe residing in Thakurgaon district [111]; itches by FMPs of villages by the Padma River in Rajshahi district [59]; gastrointestinal disorders by FMPs of Vasu Bihar village, Bogra district [108]; helminthiasis by FMPs of Balidha village in Jessore district [88]; blood dysentery, dysentery, infections by FMPs of Station Purbo Para village in Jamalpur district [89]; pain by FMPs of Shetabganj village in Dinajpur district [90]; helminthiasis, frequent urination by FMPs of Shekhertek and Badarganj villages in Rangpur district [91]; hookworm infections by FMPs of Barisal Town in Barisal district [93]; all types of pain, fever by FMPs of three villages in Panchagarh and Thakurgaon districts [95]; fever, burning sensations in the body, helminthiasis by FMPs of six villages in Greater Naogaon district [96]; skin diseases, sexual weakness by FMPs of seven villages of Ishwardy sub-district in Pabna district [97]; pain in body, blood purifier by FMPs of Babla and Terbaria villages in Tangail district [82]; sialorrhea, helminthiasis by FMPs of Paschim Shawra and Palordi villages of Gaurnadi sub-district in Barisal district [71]; fever by FMPs of Uttar Musrat Madati and Kisasat Madati villages in Lalmonirhat district [60]; waist pain by FMPs of Shonapur, Chorkulte, and Majhbari villages of Rajbari district [99]; dysentery by FMPs of Jool chotro and Janga lia villages of Tangail district [75]; jaundice by FMPs of six villages in Thakurgaon district [76]; frequent urination, diabetes by the TMPs of the Baburo, Haduga and Larma clans of the Chakma tribe residing in Rangamati district [53]; pain by the Bongshi tribe of Tangail district [54]; malarial fever, any type of stomach pain by the Tonchongya tribal community residing in Keyaju Para in Bandarban district [124]; liver problems by a FMP of Savar in Dhaka district [113]; burning sensations in the chest, salty taste in mouth when burping, flatulence, gastric pain by the Sigibe clan of the Khumi tribe residing in Thanchi sub-district of Bandarban district [47]; dental caries, abdominal pain by the Chakma tribe of Hill Tracts districts [118]; diarrhea by the Hajong community of Baromari village in Netrakona district [103]; swollen legs and blisters by the Rakhaing community of Cox’s Bazar district [52]; helminthiasis, rheumatic pain by folk medicinal herbalists in seven villages of Bhola district [105].
*Lantana camara* L.	Fever by Tripura community of Hazarikhil in Chittagong district [42].
*Alpinia nigra* (Gaertn.) Burtt.	Hemorrhoids, pain, arthritis by the Khasia tribe [11]; loss of sensation in hands and legs by the Marma tribal community residing in Naikhongchaari, Bandarban district [45]; stomach disorders by the Rakhain tribe of Chittagong Hill Tracts [121]; indigestion, stomach pain, bloating, acidity by the TMPs of Tripura tribe residing in Chittagong Hill Tracts [48]; swelling of face in cattle, cattle lice by FMPs of Shonapur, Chorkulte, and Majhbari villages of Rajbari district [99]; gastrointestinal disorders (acidity, stomach ache, diarrhea), sudden bouts of fainting, vertigo by the Tonchongya tribal community of Roangchaari sub-district of Bandarban district [49]; jaundice, gastric ulcers by the Chakma tribe of Hill Tracts districts [118].
*Curcuma longa* L.	Hypotonia (reduced muscle strength), scabies, leucoderma, to increase fertility in women, acne by the Santal tribe [4]; kala azar by the Rai tribe [10]; skin disorders by the folk medicinal practitioners (FMPs) of Jessore district [63]; wet dream, scabies, eczema by FMPs in Tangail district [82]; helminthiasis, itches by FMPs of Sylhet Division [83]; passing of semen with urine, leucorrhea by the Soren clan of the Santal tribe residing in Kannapara and Mondumala villages of Rajshahi district [84]; jaundice, diarrhea, dysentery, small pox, eczema, gonorrhea, sedative by FMPs in five villages of Boalia sub-district, Rajshahi district [116]; jaundice, skin disorders, to increase brightness of skin by FMPs of Shitol Para village, Jhalokati district [66]; gonorrhea, helminthiasis, sore throat, hepatitis, appetizer, allergy, eye disorder by FMPs of villages in Natore and Rajshahi districts [67]; acne by FMPs of Daudkandi sub-district in Comilla district [85]; snake bite by FMPs of Bheramara area in Kushtia district [68]; allergy, skin diseases, scabies, leprosy by FMPs of Rahmatpur village by the Ghaghot River, Rangpur district [59]; skin diseases by by FMPs of Vasu Bihar village, Bogra district [108]; excessive bile secretion by FMPs of Balidha village in Jessore district [88]; allergy by FMPs of Station Purbo Para village in Jamalpur district [89]; filariasis by FMPs of Shetabganj village in Dinajpur district [90]; to improve skin texture, sprain by FMPs of Daulatdia Ghat in Kushtia district [92]; arthritis, gout by FMPs of three areas of Pirojpur district [94]; jaundice, tumor, sprain, dermatitis, conjunctivitis, small pox, colic by FMPs of two villages by the Rupsha River in Bagerhat district [115]; wet dream, scabies, eczema by FMPs of Babla and Terbaria villages in Tangail district [82]; excessive sexual desire, rheumatism, leprosy, diabetes, edema by FMPs of three villages in Sreepur sub-district in Magura district [98]; skin diseases (scabies, eczema), gum diseases by FMPs of Paschim Shawra and Palordi villages of Gaurnadi sub-district in Barisal district [71]; edema by FMPs of Khulna City, Bangladesh [72]; allergy, helminthiasis in children by FMPs of Fulbaria, Baguri, and Bagh-achra villages in Jessore district [73]; blood purifier, stomach disorders by FMPs of Shonapur, Chorkulte, and Majhbari villages of Rajbari district [99]; skin diseases by FMPs of Arpara and Munshefpur villages in Jessore district [100]; helminthiasis, skin diseases, loss of appetite, to increase memory by the FMP of Kasipur village in Narayanganj district [101]; hypertension by the TMPs of the Baburo, Haduga and Larma clans of the Chakma tribe residing in Rangamati district [53]; bone fracture, sprain by FMPs of Baghbhandar, Sonahat and Kumarpara villages in Kurigram district [102]; rheumatic pain by FMPs of several villages of Faridpur and Rajbari districts [77]; cough, eczema by Tripura community of Hazarikhil in Chittagong district [42]; coughs, eczema by the Rakhaing community of Cox’s Bazar district [52]; to improve skin color, to control excessive sexual desire, rheumatism, leprosy, diabetes, edema by FMPs of Shat-tola Bazaar and Talbari villages in Bagerhat district [79]; diarrhea, dysentery by the Teli clan of the Telegu tribe [43]; to whiten complexion by healers among tea garden workers in Sreemangal [119]; blood disease by the Chakma community of Chittagong Hill Tracts [106].

Essentially, the Deb barma medicinal plants (Table 3) can be classified into four parts. First, a limited number of plants, which have many reported uses, but where there is a consensus among the various folk and tribal medicinal practitioners on the major use (even though there may be other reported uses) of the given plant. Examples of such plants are *Justicia adhatoda* (majority of healers using the plant for treatment of respiratory tract infections and particularly coughs), *Terminalia arjuna* (majority of healers using the plant for treatment of cardiovascular disorders), and *Senna alata* (most healers using the plant for treatment of skin diseases). Among the second category are plants with multiple reports of uses, but where use of the given plant for therapeutic purposes varies widely between different healers. Examples of these types of plants are *Andrographis paniculata*, *Centella asiatica*, *Alstonia scholaris*, *Asparagus racemosus*, *Terminalia bellirica*, *Terminalia chebula*, *Azadirachta indica*, *Moringa oleifera*, *Aegle marmelos*, *Curcuma longa* and *Scoparia dulcis*. The third category of plants include plants like *Aerva sanguinolenta*, *Crinum latifolium*, *Colocasia esculenta*, *Sansevieria hyacinthoides*, *Melastoma malabathricum*, *Eichhornia crassipes*, *Physalis micrantha*, *Persicaria glabra*, *Smilax macrophylla*, *Sansevieria hyacinthoides*, *Garcinia cowa* and *Lantana camara*, whose reported uses by FMPs or TMPs are less in number. In fact, *Physalis micrantha*, *Persicaria glabra*, *Smilax macrophylla*, *Sansevieria hyacinthoides*, *Garcinia cowa* and *Lantana camara* each have only one reported ethnomedicinal use in Bangladesh besides their use by the Deb barma healer. The fourth category includes plant like *Pouzolzia zeylanica*, whose use appears to be unique to the Deb barma healer in the sense that its ethnomedicinal uses in Bangladesh are yet to be reported to the best of our knowledge. Thus use of this plant by the Deb barma can be considered novel.

A number of medicinal plants used by the Deb barma healer had at least one reported similar ethnomedicinal use by FMPs or TMPs of Bangladesh. To cite a few instances, *Andrographis paniculata* used by the Deb barma healer for treatment of malaria has been reported to be used for treatment of malarial fever by the Bauri tribal community [61]. *Justicia adhatoda* has been reported to be used for treatment of skin infections by the Kanda tribe [51] and tuberculosis by FMPs in Tangail district [62]; the plant was used by the Deb barma healer to treat skin infections as well as tuberculosis. *Justicia gendarussa*, used by the Deb barma healer for treatment of coughs and malaria, has been reported to be used for treatment of coughs by the Naik clan of the Rajbongshi tribe [46]. *Aerva sanguinolenta*, used by the Deb barma healer for treatment of cuts and wounds, reportedly has similar use [61]. The same applies for the use of *Crinum latifolium* for treatment of bloating in cattle by the Deb barma healer; the plant has been reported to be used for indigestion in cattle (which can lead to bloating) by the Khasia tribe of Sylhet district [11]. However, some uses are unique to the Deb barma healer, being not reported from elsewhere in Bangladesh. These include use of *Alstonia scholaris* for treatment of formation of whitish layer on tongue, use of *Terminalia arjuna* for treatment of burning sensations during urination, use of *Phyllanthus emblica* for treatment of paralysis, use of *Sansevieria hyacinthoides* for treatment of snake bite and as snake repellent, the use of *Garcinia cowa* for treatment of coughs and colds, and use of *Melastoma malabathricum* for treatment of cuts and wounds, to cite a few examples.

### Relevance of uniqueness of several Deb barma medicinal plants and their uses

The uniqueness of use of some medicinal plants for therapeutic purposes by the Deb barma healer suggests that these plants (like *Physalis micrantha*, *Persicaria glabra*, *Smilax macrophylla*, *Sansevieria hyacinthoides*, *Garcinia cowa*, *Pouzolzia zeylanica* and *Lantana camara*) be scientifically examined for their relevant pharmacological activities, which can validate their traditional uses. A number of plants used by the Deb barma healer have been shown earlier to have scientific validations in their traditional uses. Such scientific studies and validations can be important from at least three view points. First, it establishes confidence among scientists, doctors as well as the general people that traditional medicine can be useful and safe to use. Second, such scientific studies can lead the way to possible discovery of lead compounds and drugs from the medicinal plants. Third, the rural people can benefit a lot from using these plants for therapeutic purposes instead of allopathic drugs, which may be costly for them, or like in most rural areas of Bangladesh, inaccessible, due to absence of modern doctors and health facilities. As such, use of traditional medicine can lower the medical costs (because in general herbal drugs are cheaper than allopathic drugs in Bangladesh) and so benefit the poorer segments of the population, who form the majority of people in Bangladesh and other developing countries.

## Conclusion

The Deb barma clan is a comparatively small clan of the Tripura tribe with its current total population at only 135 members in Srimangal of Moulvibazar district, Bangladesh. Their ethnomedicinal practices have not been previously reported although they have their own traditional medicinal system and their own traditional healer. Interviews with the healer and adult members of the clan indicated that they believed diseases to occur from the curses of a particular evil god, or caused by evil spirits and demons. Their traditional methods of curing included oral or topical use of medicinal plants, wearing of amulets, appeasement of the evil god through worship and offerings, and treatment of black magic-induced disease with counter-black magic.

A survey of the existing literature showed that the use of a number of plants by the traditional healer for treatment of specific ailments could be scientifically validated based on the reported pharmacological activities of the plants used. The tribal medicinal system of the Deb barma clan showed a notable similarity with Ayurvedic form of treatment (which is considered the most ancient form of treatment within the Indian sub-continent) in terms of plant used and ailments treated. Considering that the two systems had probably interacted with each other for at least two thousand years, it is very much plausible that each system could have influenced the other. However, medicinal uses of a number of the plants differed between the Deb barma clan and other tribes of Bangladesh, the medicinal practices of which have been documented. The differences indicate the importance of documenting the medicinal practices of as many tribes as possible to obtain an overall view of the diverse uses of any given plant species.

Our interviews further suggested that in recent years, the Deb barma clan members may have started to prefer allopathic system more than their traditional medicinal system. If this happens, the ethnomedicinal wisdom of the Deb barma clan may be lost forever, if not documented. Since already the usage of a number of their traditional medicinal plants has been validated through scientific research, it is important that the yet to be studied plants be examined scientifically as to their pharmacological properties and their phytochemical constituents. Such studies can be beneficial to human beings if new and more efficacious medicines can be discovered from these plants.

## Competing interests

The authors declare that they have no competing interests.

## Authors’ contributions

MHK, NH, MMR, MAR, JAK, NTH, MRQB, and SMM participated and completed the ethnomedicinal survey under the supervision of MR and submitted an initial report of the survey. MR and RJ analyzed the data and wrote the manuscript. All authors edited the manuscript and read and approved the final manuscript.
